# Heterogeneous Wettability‐Based Construction of Two‐Phase Interfaces for Underwater Reversible Adhesion

**DOI:** 10.1002/advs.202524235

**Published:** 2026-03-02

**Authors:** Xiaokai Li, Yonghui Zhang, Yongxin Li, Jiahao Zhang, Yuheng Li, Zhengyu Li, Jianwu Wang, Yaochen Lv, Yanan Wang, Jiyu Liu, Xin Liu, Huanxi Zheng

**Affiliations:** ^1^ State Key Laboratory of High‐performance Precision Manufacturing Dalian University of Technology Dalian P. R. China; ^2^ State Key Laboratory of Woody Oil Resources Utilization Northeast Forestry University Harbin P. R. China; ^3^ College of Mechanical and Electrical Engineering Northeast Forestry University Harbin P. R. China

**Keywords:** heterogeneous wettability surface, on‐demand detachment, superhydrophilic, superhydrophobic, underwater adhesion

## Abstract

Underwater smart adhesives with switchable adhesion have attracted significant attention for applications in marine engineering and biomedical fields. However, achieving both robust adhesion and rapid on‐demand detachment in aquatic environments remains a critical challenge. Inspired by the diving bell spider, we propose a design strategy based on heterogeneous wettability surfaces to achieve switchable adhesion between two surfaces. The heterogeneous wettability surface design confines the oil phase within air cavities formed in superhydrophobic (lipophilic) regions, thereby creating annular oil rings, which establish stable oil/water interfaces that spatially isolate the internal water bridge from the external aquatic environment. The Laplace pressure difference generated at the oil/water interfaces induces robust adhesion between surfaces. In addition, the adhesion strength can be linearly enhanced by constructing multiple, uniformly arranged oil/water interfaces. Rapid on‐demand separation can also be achieved through controlled electrolysis of the internal water bridge by applying voltage between the surfaces. Finally, we demonstrate the practical applicability of this adhesive by integrating it into an unmanned underwater vehicle (UUV) for efficient underwater anchoring and ship‐hull hitchhiking, highlighting its broad application potential in underwater engineering scenarios.

## Graphical abstract

The design employs heterogeneous wettability surfaces that construct stable oil‐water interfaces to achieve robust and rapid reversible adhesion. Linear enhancement of adhesion strength is realized through multi‐interface patterning. This adhesive enables rapid on‐demand detachment within five seconds via voltage‐induced electrolysis, offering a level of environmental adaptability and performance scalability not previously achievable.

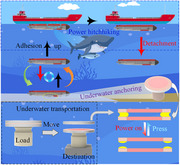

## 1 Introduction

In recent decades, the rapid advancement of marine engineering and biomedicine has spurred an urgent demand for switchable smart adhesives capable of functioning in underwater environments [1, 2, 3, 4, 5]. Conventional adhesives perform well in dry environments, but often fail in underwater environments due to the plasticizing effect of water, the barrier of interfacial hydrated film, and ionic interference [6, 7]. In nature, numerous marine organisms have evolved the ability to form switchable adhesion with underwater materials, such as mussels [8, 9], sandcastle worms [10], and barnacles. Inspired by these organisms, researchers have developed various underwater chemical adhesives with stimulus‐responsive, reversible adhesion capabilities [11, 12, 13, 14, 15, 16]. Although such adhesives have achieved stimulus‐responsive adhesion in underwater environments, most rely on chemical synthesis strategies involving covalent bond formation, resulting in complex preparation processes and slow reversible response rates. These limitations severely constrain the practical application of such reversible underwater adhesives. In contrast, octopuses and starfish have evolved more sophisticated and multifunctional underwater attachment systems that integrate synergistic mechanisms such as adsorption, interfacial sealing, soft tissue mechanics, and active control. Inspired by these biological attachment mechanisms, researchers have developed multiple switchable adhesive devices and materials [17, 18, 19, 20, 21, 22]. While such adhesion systems eliminate complex chemical synthesis pathways, they present new technical challenges, including stringent manufacturing precision requirements and cumbersome preparation processes.

In air, a thin liquid film sandwiched between the surfaces of two objects provides an efficient physical adhesion mechanism based on capillary action. This natural mechanism, requiring no complex chemical synthesis, has been widely applied in the field of adhesion. For example, Vogel et al. [23] established a reversible liquid bridge between a device and a target object by controlling the stretching and retraction of droplets on the device surface, thereby achieving reversible adhesion to the object. Li et al. [24] reported a novel oil‐carrying shape memory polymer microcolumn with intelligent controllability. The microcolumn could reversibly switch between tilted and upright states while altering the distribution of captured oil to modulate capillary forces, thus achieving reversible adhesion to various objects. Similarly, Ma et al. [25] designed a honeybee‐tree frog‐inspired hierarchical patterned surface that effectively dispersed moisture at the contact interface in humid environments, forming tight contact and achieving strong wet adhesion through capillary action, which could be applied to bio‐signal detection on moist human surfaces. These studies demonstrated the versatility of capillary‐based adhesion in air or humid environments; nevertheless, this mechanism relied critically on the presence of a gas‐liquid interface to generate Laplace differential pressure. In underwater environments, the absence of a gaseous phase eliminates the gas/water interface, thereby preventing the formation of effective Laplace differential pressure and rendering the mechanism ineffective [26]. To overcome this limitation, we previously [27] employed a superhydrophobic surface to trap gas and form stable air cavities, thereby isolating the internal water bridge from the external aqueous environment to achieve reversible adhesion between two surfaces. Wang et al. [28] similarly utilized surface superhydrophobicity, employing bubbles as an adhesive to enable reversible adhesion over large areas. These approaches provide a more reliable mechanism for reversible adhesion between two surfaces through re‐establishing the Laplace pressure difference. Additionally, Weinstein et al. [29] constructed a syringe‐based pump system capable of reversibly injecting air into the adhesive head to create an air capillary bridge underwater, which achieved controllable regulation of surface adhesion in aquatic environments.

Current underwater capillary reversible adhesion mechanisms primarily rely on constructing sealed air cavities to exploit the Laplace pressure difference generated at the gas/water interface, thereby achieving controllable adhesion. However, the inherent fragility of the gas/water interface makes it prone to failure in complex underwater environments [30], particularly under significant fluctuations or in the presence of contaminants. Especially for strategies relying on superhydrophobic surfaces to trap gas and form cavities, adhesion performance often degrades significantly or fails entirely when exposed to real‐world environmental challenges (Contaminants readily accumulate within the microstructure of superhydrophobic surfaces, causing the loss of hydrophobicity, as shown in Figure S1b,e), severely limiting their practical applications. Therefore, it is of great significance to develop a novel reversible adhesive strategy that can maintain robust and stable performance across diverse and challenging practical environments.

Here, inspired by the ability of diving bell spiders to retain air cavities underwater, we report a novel, green, stable, and rapidly detachable adhesive that can well address these challenges by replacing the conventional air/water interface with a stable oil/water interface via a heterogeneous wettability surface design. This design locally confines an oil phase within cavities formed by superhydrophobic (lipophilic) regions to create annular sealing rings. These rings construct stable oil/water interfaces that isolate internal water bridges from the external aqueous environment, enabling robust adhesion between the two surfaces via the Laplace pressure difference. In contrast to the instability of air/water interfaces, the oil/water interfaces demonstrate superior environmental adaptability (Figure S1c,f) and retain structural integrity during cross‐media transitions, thereby significantly expanding the application scenarios of capillary adhesion. Interestingly, rapid on‐demand detachment can be achieved by inducing controlled electrolysis of internal water bridges through applying a voltage between the two surfaces. Furthermore, our smart capillary adhesive is compatible with various rigid and flexible substrates, enabling precise underwater object transport and cross‐medium transfer (water‐to‐air). Finally, we demonstrate the integration of this design into underwater unmanned vehicles for efficient underwater anchoring and power hitchhiking. This work provides new insights into the design of underwater bionic adhesion systems and has broad application prospects in fields such as deep‐sea exploration, underwater robotics, and cross‐medium transportation.

## 2 Results and Discussion

### 2.1 Design Concept and Working Principle

**FIGURE 1:**
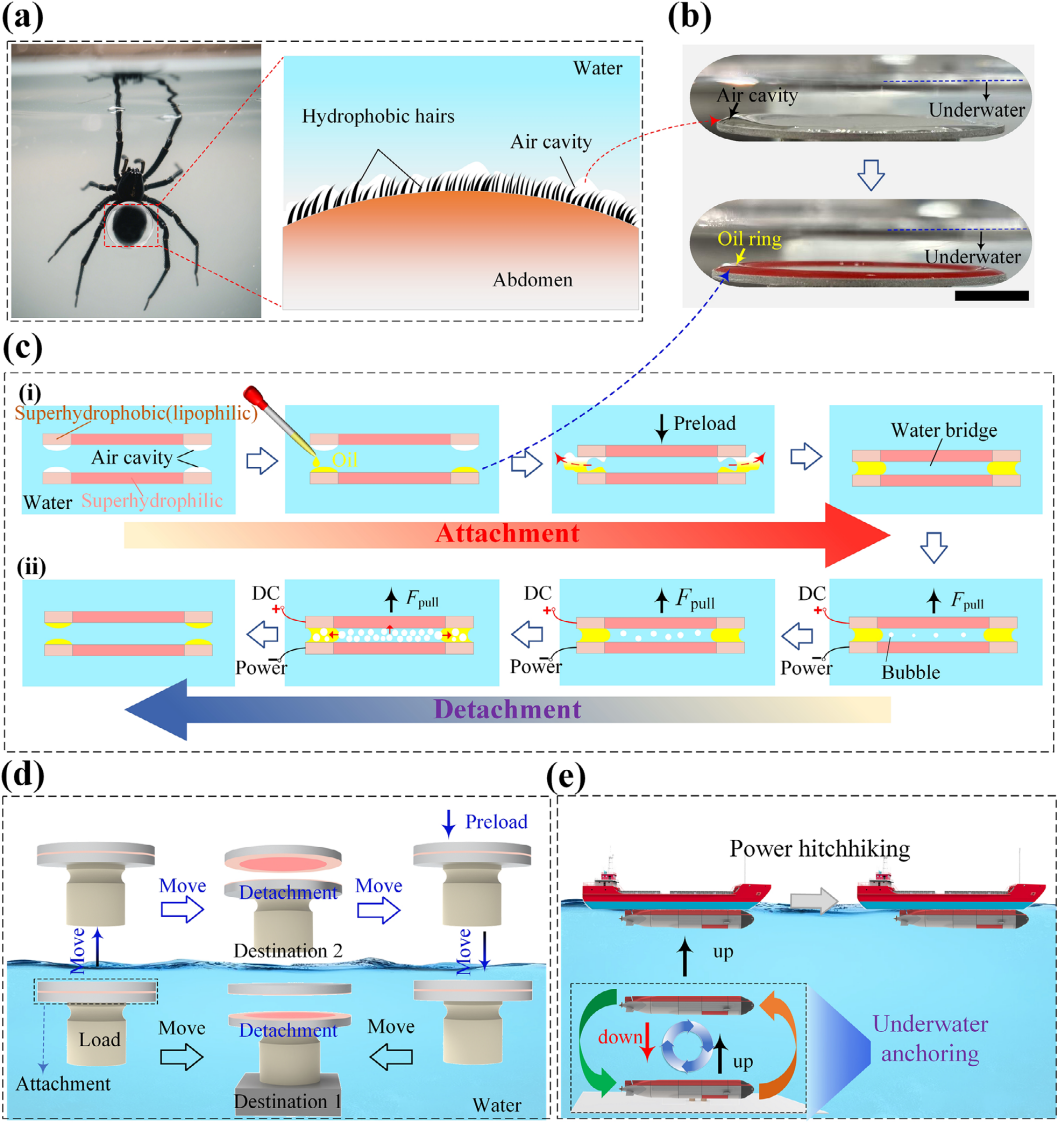
Working principle and applications of the underwater reversible capillary adhesion. (a) The mechanism by which diving bell spiders form air chambers underwater. (b) Optical pictures of an air cavity formed by a superhydrophobic region on a heterogeneous wettability surface underwater, and peanut oil filled in the air cavity. Here, the scale bar is 1 cm, the substrate is an Al plate, and the oil phase has undergone dyeing treatment. (c) The working principle of underwater capillary attachment and detachment. (d), (e) Applications.

Inspired by the hydrophobic abdominal hairs of the diving bell spider that form air cavities underwater (Figure 1a), we design heterogeneous wettability surfaces to achieve reversible underwater adhesion. First, the heterogeneous wettability surfaces are prepared by laser etching and low surface energy modification (Figure S2). Their superhydrophobic (lipophilic) regions mimic the spider's survival mechanism, forming spatially confined air cavities underwater to provide a spatial foundation for the filling of oil‐based substances (Figure 1b). In contrast, the superhydrophilic regions are completely wetted by water [31, 32, 33, 34, 35] (Relevant wetting data are shown in Figure S3 and Table S1). As illustrated in Figure 1c (i), prior to adhesion of two heterogeneous wettability surfaces, a quantitative oil phase is first introduced into the cavities formed within the superhydrophobic region of one surface, creating a continuous and stable oil ring. When these surfaces are brought into proximity under an external preload, the pre‐filled oil phase spreads into the vacant superhydrophobic cavities of the other surface due to the lipophilic properties of the superhydrophobic regions. As the load increases, air trapped within the unfilled superhydrophobic cavities is gradually expelled from the cavity edges. Simultaneously, the aqueous phase within the superhydrophilic regions of both surfaces comes into contact and merges, forming a water bridge connecting the two surfaces. Ultimately, a well‐defined structure emerges where the oil ring encapsulates the water bridge, thereby establishing a clear and stable oil‐water interface (Figure S4). Furthermore, as shown in Figure 1c (ii), rapid on‐demand detachment can be achieved by applying a voltage between surfaces to trigger controlled electrolysis of the internal water bridge, generating bubbles consisting of H_2_ and O_2_ (2H_2_O = 2H_2_↑+O_2_↑). Thus, the heterogeneous wettability surface‐based adhesives demonstrate a natural capillary adhesion mechanism that avoids complex chemical synthesis, showing promising application potential in underwater transportation, cross‐medium transfer, and unmanned underwater vehicles navigation (Figure 1d,e).

### 2.2 Performance Characterization of Adhesion Force

**FIGURE 2:**
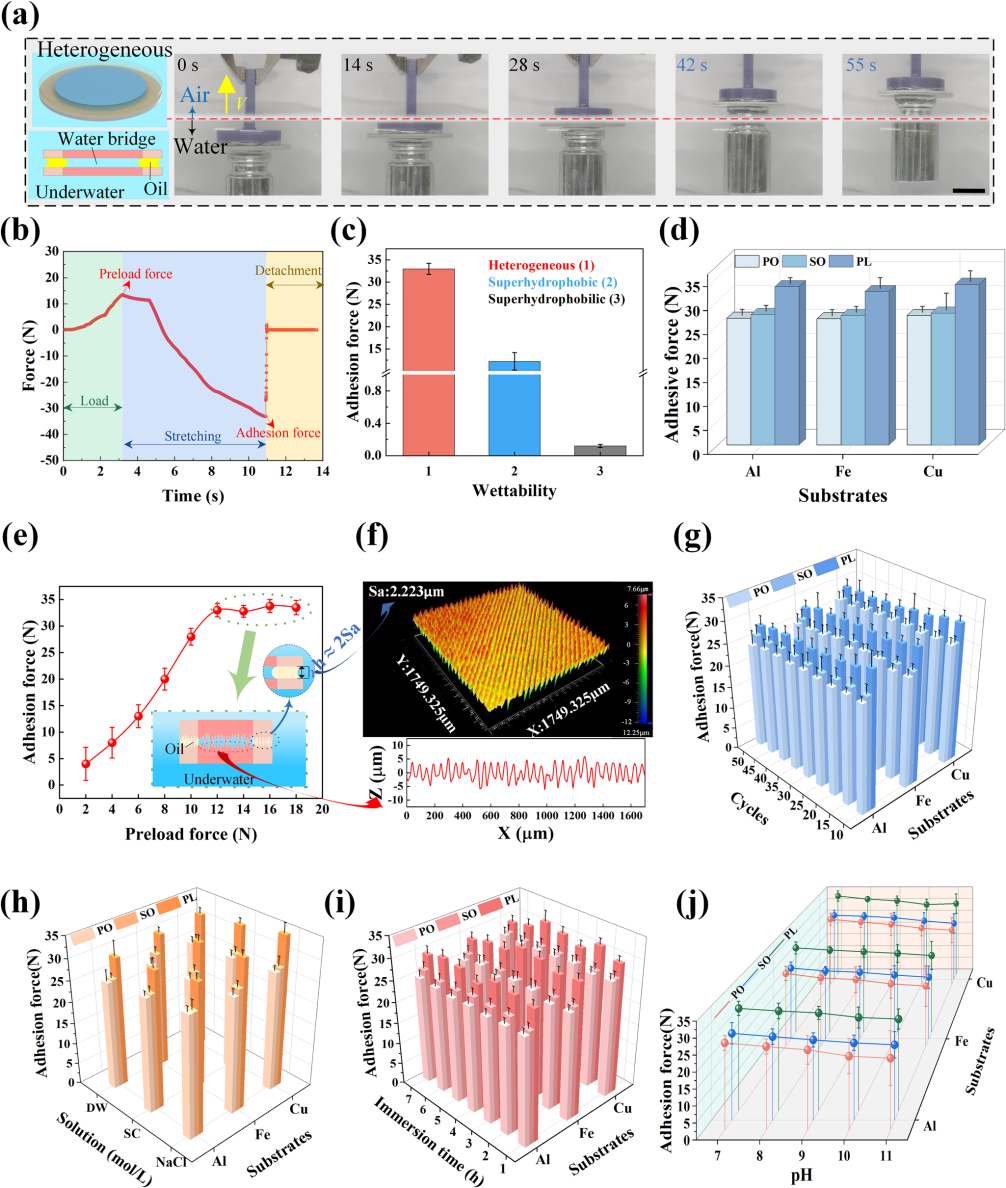
Characterizations of underwater capillary adhesion properties. Here, the radius (*R*_SHL_) of the superhydrophilic region is 1.5 cm, and the radius (*R*) of the substrate is 2 cm. (a) Selected optical pictures of heterogeneous wettability surfaces during transfer from water to air with a load of 150 g. Here, the scale bar is 2 cm, the substrate is an Al sheet, and the filler oil is paraffin liquid. (b) Dynamic change of forces during the adhesion‐detachment process of two heterogeneous wettability surfaces. Here, the substrate is an Al sheet, the filler is paraffinic liquid, the preload force is 13.4 N, and the adhesion force is 33.2 N. (c) The adhesion force of surfaces with different wettability underwater. (d) The influences of different substrates and filling oils in the air cavity on adhesion force. Here, SO is silicone oil, PO is peanut oil, and PL is paraffinic liquid. (e) The effect of preload force on adhesion force. (f) 3D morphology and cross‐section of heterogeneous wettability surfaces. (g) Changes in adhesion force during 50 cycles. The maximum adhesion force decreases by only about 10% after 50 cycles. (h–j) The effects of different solutions (h), underwater immersion time (i), and pH (j) on adhesion force. The error bars in each figure represent deviations from five repeated measurements.

As shown in Figure 2a and Movie S1, the heterogeneous wettability‐based underwater adhesives maintain stable adhesion in both aquatic and aerial environments, enabling reliable water‐air cross‐media transfer of 150 g loads. From the perspective of surface wettability characteristics, superhydrophilic surfaces in underwater environments are preferentially and completely wetted by the aqueous phase. As a result, the oil phase cannot readily spread over or adhere to these surfaces, preventing the formation of an oil‐water interface. In contrast, superhydrophobic surfaces form air cavities underwater, which not only prevent the direct contact between the aqueous phase and the solid surface but also provide an ideal space for oil phase filling. However, due to their homogeneous wettability, such a surface cannot form multi‐region synergistic oil‐water interface structures like those on heterogeneous wettability surfaces. To compare these differences, homogeneous superhydrophobic and superhydrophilic surfaces are also tested under identical test parameters. In contrast, the homogeneous superhydrophobic surfaces exhibit adhesion failure during load lifting, while the homogeneous superhydrophilic surfaces are completely wetted by water, causing oil‐phase attachment failure and loss of load‐bearing capacity (Figure S5). This phenomenon indicates the critical role of the oil ring in encapsulating the internal water bridge. To further evaluate the adhesion performance and quantitatively compare different surface designs, adhesion forces between two surfaces with different wettability (heterogeneous, homogeneous superhydrophobic, homogeneous superhydrophilic) are measured and analyzed. For adhesion force measurement, with the lower surface immobilized and the upper surface displaced vertically at 330 µm/s, force‐time curves during the adhesion‐detachment process are recorded, and the adhesion forces between different surfaces can be determined from these curves (Figure 2b; Figure S6). As shown in Figure 2c, the adhesion force between the two heterogeneous wettability surfaces is approximately 33.2 N, roughly 2.7 times and 276 times higher than those of homogeneous superhydrophobic surfaces (12.2 N) and superhydrophilic surfaces (0.12 N), respectively. Notably, the adhesion force of the superhydrophobic surface is considerably higher than that of the superhydrophilic surface. This difference stems from the ability of the superhydrophobic surface to generate a Laplace pressure difference that drives adhesion, whereas the superhydrophilic surface cannot. These results confirm that the heterogeneous wettability design, by precisely regulating the surface wettability distribution, can construct a stable multi‐regional oil‐water interface system, thereby significantly enhancing the capillary adhesion force.

We further investigate the effects of substrate materials and the types of oil used to fill cavities on the adhesion properties. As shown in Figure 2d, substrate materials have no significant influence on the adhesion force, whereas filling the cavities with paraffin liquid (PL) yields higher adhesion forces compared to peanut oil (PO) or silicone oil (SO). This difference can be attributed to the higher surface tension of the PL‐water interface relative to the PO‐water and SO‐water interfaces. On the other hand, the adhesion forces remain substantial in each case, which demonstrates the compatibility of our underwater capillary adhesive with different substrates and oil types, showing promising practical application prospects. Figure 2e illustrates the effect of preload force on the underwater adhesion force, which gradually increases with the preload force, but stabilizes at ≈ 33 N when the preload force reaches ≈ 12 N. This indicates that the thicknesses of the oil ring and water bridge have reached the surface structural roughness scale at this point (Figure 2f; Figure S7). Notably, our reversible underwater capillary adhesive combines durable adhesion with reusability, demonstrating stable tolerance to diverse loads in underwater environments for over 72 consecutive hours (Figure S8) and showing no significant degradation after 50 cycles (Figure 2g). As shown in Figure S9, even after multiple attachment‐detachment cycles, the oil phase on the superhydrophobic surface remains stably anchored without detachment or dispersion, effectively maintaining the integrity of its overall distribution structure.

Considering real‐world underwater application environments, we measure and analyze the adhesion forces in various aqueous conditions (Figure S10). As shown in Figure 2h, the adhesion force in NaCl solution and artificial seawater (sea crystal solution, SC) exceeds that in deionized water (DW). This enhancement is due to the hydration effect of Na^+^ and Cl^−^ ions in the solutions, where water molecules can rearrange to form ion‐dipole interactions [36, 37]. As a result, the cohesive force between water molecules increases, raising interfacial tension above that of pure water. As shown in Figure 2i, the underwater immersion time has no significant negative effect on the adhesion force (the oil phase is tightly adsorbed in the superhydrophobic region and does not loosen with increasing immersion time, Figure S11). Furthermore, regardless of the oil phase filled in the gas cavity, the adhesion force of the adhesives decreases with increasing pH (Figure 2j), which is due to the saponification reaction of oily substances under alkaline environments, generating surfactants that diffuse into the oil‐water interface and reduce interfacial tension [38]. Among these oil phases, paraffin oil exhibits better resistance to alkaline degradation than peanut oil and silicone oil, maintaining more stable adhesion performance under high‐pH conditions.

### 2.3 Mathematical Modeling of Adhesion Force

**FIGURE 3:**
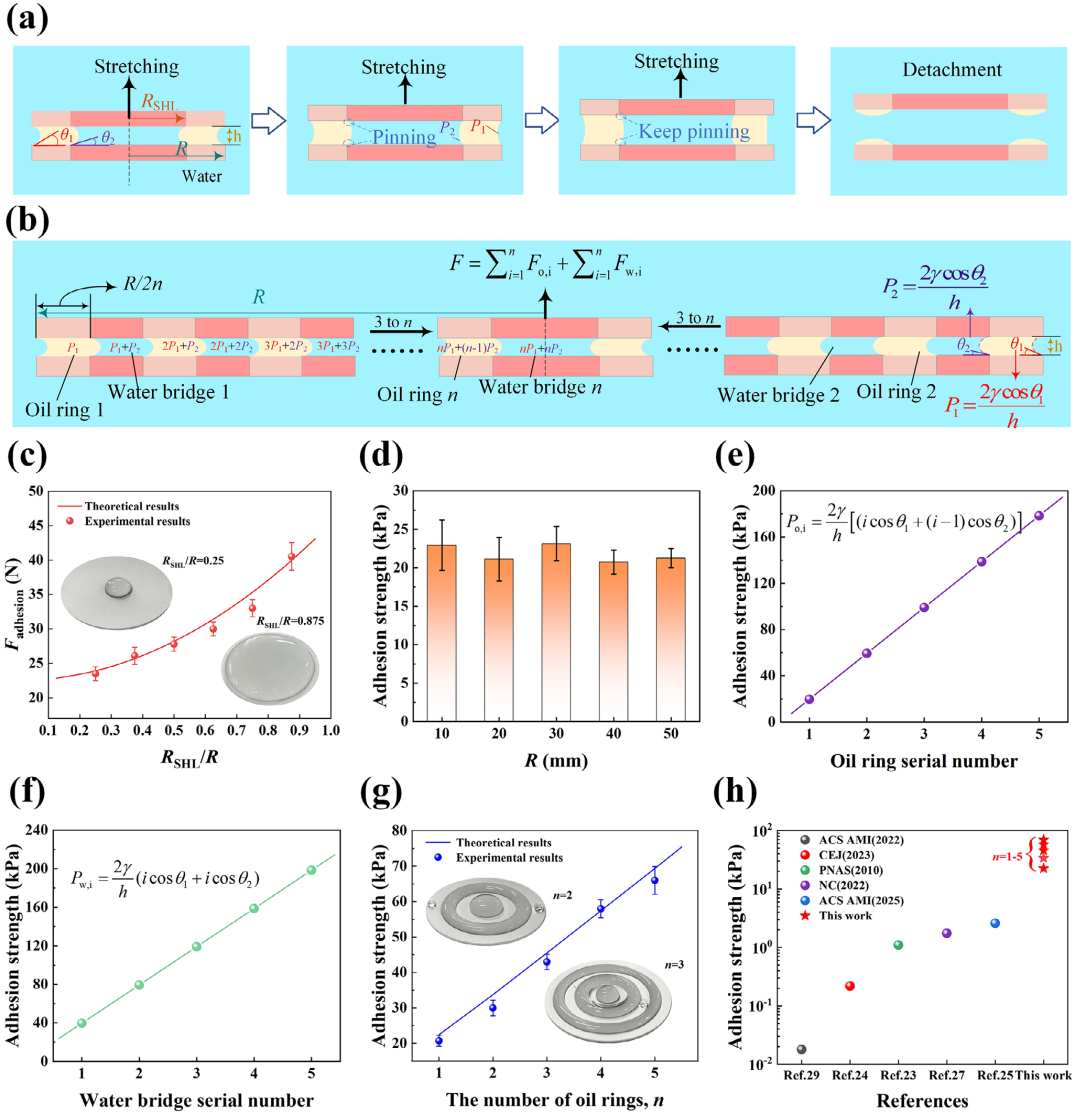
Mathematical model analysis and performance comparison of adhesion force. (a) Schematic diagram of the dynamic changes of oil and water bridges during the stretching of two heterogeneous wettability surfaces. (b) Schematic diagram of the adhesion state of two heterogeneous wettability surfaces under multiple oil rings. (c) The effect of *R*_SHL_/*R* on the adhesion strength of the experimental value and the mathematical model. Here, *R* = 2 cm. (d) The effect of *R* on adhesion strength. Here, *R*_SHL_/*R* is 0.5, and the adhesion strength is stable at ≈ 22.5 kPa (theoretical value). (e) Adhesion strength of different oil rings under a multi‐ring system. (f) Adhesion strength of different water bridges under a multi‐ring system. In (e) and (f), the theoretical results are calculated with paraffin liquid as the filler. (g) The effect of the number of oil rings on the adhesion strength. (h) Comparison of our adhesive with other adhesives prepared based on capillary principles. The filler in (c), (d), (g), and (f) is paraffin liquid, and the substrate is an Al sheet. The error bars in each figure represent deviations from five repeated measurements.

The above wettability comparison experiments demonstrate that the oil ring encapsulating the water bridge can significantly improve the performance of underwater capillary adhesives. Therefore, it is necessary to conduct an in‐depth analysis of the working mechanism of the underwater capillary adhesives. Figure 3a illustrates the dynamic evolution of the oil‐water interface during stretching. When the upper surface is lifted under external force, the oil remains anchored in the superhydrophobic region, effectively resisting the external stretching force due to the complete wetting of the superhydrophilic region. As the external stretch force continues to increase, the adhesion state eventually fails (exceeding the tolerance limit of Laplace pressure difference) at the instant the oil begins to move toward the inner superhydrophilic region, resulting in the detachment of the two surfaces.

Based on the above analysis, we establish mathematical models to predict underwater adhesion force. The Laplace pressure difference formed between the oil ring and the external water environment can be calculated as follows:

(1) $$\Delta P_{1}=\gamma \left(1/R+2\cos\theta_{1}/h\right)$$

The adhesive force contribution of the oil ring can be obtained by the following equation:

(2) $$F_{1}=\Delta P_{1}A_{1}=\pi \gamma \left(R^{2}-R_{\mathrm{SHL}}^{2}\right)\left(1/R+2\cos\theta_{1}/h\right)$$

where γ is the surface tension of the oil‐water interface, *A*_1_ is the area of the oil ring, and θ_1_ is the contact angle of the oil on the superhydrophobic surface. *h* is the thickness of the oil ring or water bridge, *R* is the sample radius, and *R*_SHL_ is the radius of the superhydrophilic region.

Meanwhile, the Laplace pressure difference formed between the internal water bridge and the external water environment can be amplified as the accumulated Laplace pressures at the internal and external oil‐water interfaces:

(3) $$\Delta P_{2}=\gamma \left[1/R_{\mathrm{SHL}}+1/R+2\left(\cos\theta_{2}+\cos\theta_{1}\right)/h\right]$$

The adhesion force contribution of the internal water bridge can be expressed as follows:

(4)

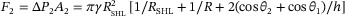

where *A*_2_ is the area of the internal water bridge, θ_2_ is the contact angle of water in the superhydrophilic region (≈ 0°). Furthermore, considering that *R* ≫ *h*, the total adhesion force can be calculated as follows [39, 40, 41] (Note S1):

(5) $$F_{\mathrm{total}}=F_{1}+F_{2}=\frac{2\gamma \pi R^{2}}{h}\left[{\left(\frac{R_{\mathrm{SHL}}}{R}\right)}^{2}\cos\theta_{2}+\cos\theta_{1}\right]$$

Correspondingly, underwater adhesion strength can be calculated as:

(6) $$\frac{F_{\mathrm{total}}}{\pi R^{2}}=\frac{2\gamma }{h}\left[{\left(\frac{R_{\mathrm{SHL}}}{R}\right)}^{2}\cos\theta_{2}+\cos\theta_{1}\right]$$

As shown in Equation (5), when *R* remains constant, the adhesion strength increases parabolically with increasing *R*_SHL_/*R*, and this trend is highly consistent with experimental results (Figure 3c; Figure S12a). In addition, Equation (6) indicates that when *R*_SHL_/*R* remains constant, the adhesion strength is independent of *R*. Experimental results (Figure 3d; Figure S12b) validate the mathematical model, which broadens the selectable range of the substrate dimensions. More importantly, the establishment of mathematical models further corroborates the analysis results regarding the influence of parameters such as preload force and solution type on adhesion force.

The wettability comparison experiments and the theoretical mathematical model analysis prove that the encapsulation of the oil ring to the internal water bridge can significantly improve the adhesion strength. Based on this, we construct *n*‐times uniform arrays with heterogeneous wettability (where *n* represents the number of air cavities and oil rings) to obtain uniformly distributed and alternatively arranged air cavities and oil rings with a width of *R*/2*n*, and further establish a theoretical model of multiple oil rings to predict the adhesion force (Figure 3b). The adhesion strength of the *i*‐th oil ring and water bridge can be respectively calculated as follows:

(7) $$\Delta P_{o,i}=\left(2\gamma /h\right)\left[\left(i\cos\theta_{1}+\left(i-1\right)\cos\theta_{2}\right)\right]$$

(8) $$\Delta P_{w,i}=\left(2\gamma /h\right)\left[\left(i\cos\theta_{1}+i\cos\theta_{2}\right)\right]$$

where *i* denotes the centripetal increment serial number of the oil ring or water bridge. Thus, in the multi‐ring system, the internal oil rings and water bridges are encapsulated, causing a pressure difference gradient between the water bridge/oil ring and the external water environment to increase with approach toward the center (as shown in Figure 3e,f). Consequently, the overall adhesion strength can be calculated as follows (see Methods):

(9) $$\begin{array}{cc} \frac{F_{n}}{\pi R^{2}} & =\frac{\sum_{i=1}^{n}F_{o,i}+\sum_{i=1}^{n}{F_{w,}}_{i}}{\pi R^{2}}\\ & =\frac{2\gamma }{h}\left[\left(\frac{3n+1+2n^{2}}{6n}\right)\cos\theta_{1}+\left(\frac{4n^{2}-1}{12n}\right)\cos\theta_{2}\right] \end{array}$$

where *F*_o_ and *F*_w_ are the adhesion forces of the oil ring and water bridge, respectively. As shown in Figure 3g and Figure S12c, the adhesion strength exhibits a linear growth trend with increasing *n*, which is highly consistent with the theoretical prediction. When *n* = 5, the adhesion strength is about 70 kPa, exceeding most existing adhesives based on the capillary principle (Figure 3h). Notably, when *n* is increased to 350, this natural adhesive, involving no chemical synthesis, can achieve an adhesion strength of ≈ 4220 kPa, comparable to reported state‐of‐the‐art chemical adhesives (Figure S13). However, if the air cavity width is compressed to a critical threshold due to the sharp increase in the number of rings, additional effects such as adhesion between air cavities may occur. Excessively small inter‐cavity spacing can lead to interference in the interfacial tension field, potentially causing the oil phase to cross predefined distribution boundaries and permeate into adjacent air cavities, thereby exacerbating the disordered spreading of the oil phase.

### 2.4 Underwater Transport and Detachment Mechanism

**FIGURE 4:**
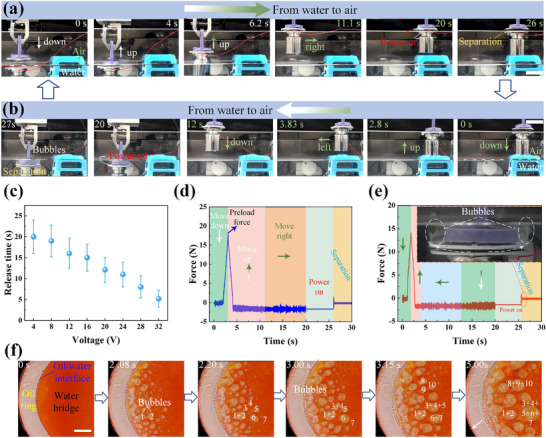
Underwater capillary adhesive for load‐bearing cross‐media transfer and on‐demand detachment. (a) The selected images show the pick‐up of a 150 g load from water and release into the air. Scale bar: 2 cm. (b) The selected images show the pick‐up of a 150 g load from air and release into water. Scale bar: 2 cm. (c) Effect of voltage on release time. Here, the error bar is the deviation of five measurements. (d) Data collected by the force sensor during the transfer of the load from water to air. (e) Data collected by the force sensor during the transfer of the load from air to water. Here, the built‐in figure shows a large number of bubbles emerging from the interior of two heterogeneous wettability surfaces at the moment of detachment. Scale bar: 1 cm. (f) Selected images showing the electrolysis process of the internal water bridge. Here, the voltage is 30 V, the scale bar is 5 mm, and the visualization window at the top is a glass plate with ITO coating that exhibits heterogeneous wettability.

Underwater load‐bearing experiments demonstrate that our underwater reversible adhesive based on heterogeneous wettability exhibits durable adhesion underwater (capable of sustaining a 300 g load for over 72 h underwater). On the basis of the robust load‐bearing capacity, we further investigate its stability during dynamic motion. Remarkably, the adhesive can precisely move a 150 g load to any predefined position, including across the water‐air interface (Figure 4a,b; Movie S2). Additionally, as shown in Figure 4c, the release time gradually shortens as the voltage increases. When the voltage reaches 32 V, rapid release can be achieved within approximately 6 s. Data collected by the force sensor during the water‐air bidirectional transport process confirms the stability of the adhesion state (Figure 4d,e). This capillary adhesive, which combines cross‐media adaptability and rapid detachment, is currently difficult to achieve with commercial adhesives.

Additionally, at the instant of detachment between two heterogeneous wettability surfaces, a large number of bubbles generated by electrolysis can be clearly observed emerging from the interior (built‐in diagram in Figure 4e). To elucidate the dynamic mechanism of the water bridge under voltage‐induced electrolysis and its impact on adhesion forces, indium tin oxide (ITO) conductive glass with heterogeneous wettability is used as a top visualization window to study the electrolysis process of the water bridge (Figure S14 and Movie S3). As shown in Figure 4f, during the initial electrolysis stage, a large number of tiny bubbles form inside the water bridge. As electrolysis continues, these tiny bubbles gradually coalesce, reducing the contact area between the water bridge and the substrate, thus decreasing the adhesion force. Moreover, since the oil ring encapsulates the internal water bridge, the accumulation of bubbles weakens the isolation effect of the oil ring, leading to further decline in adhesion. When the adhesion force generated by the oil ring and the water bridge is lower than the load weight, the adhesion state collapses.

### 2.5 Applications

In ocean engineering and military applications, unmanned underwater vehicles (UUVs) are increasingly becoming critical tools for performing tasks such as exploration and monitoring [42]. However, when undertaking long‐distance cruising or stationary monitoring missions, UUVs still face significant bottlenecks in terms of energy efficiency and positional stability. Traditional propulsion methods consume enormous amounts of energy, thus limiting range and endurance, and maintaining a fixed position in turbulent flows requires continuous electricity supply, further exacerbating energy consumption [43]. Conventional mechanical clamping devices generally require highly specialized surface designs, with the clamping or releasing process not only consuming significant energy but also causing disturbance to the surrounding environment. Vacuum adsorption methods, on the other hand, impose stringent requirements on sealing surfaces, making it difficult to achieve effective adhesion on porous surfaces.

**FIGURE 5:**
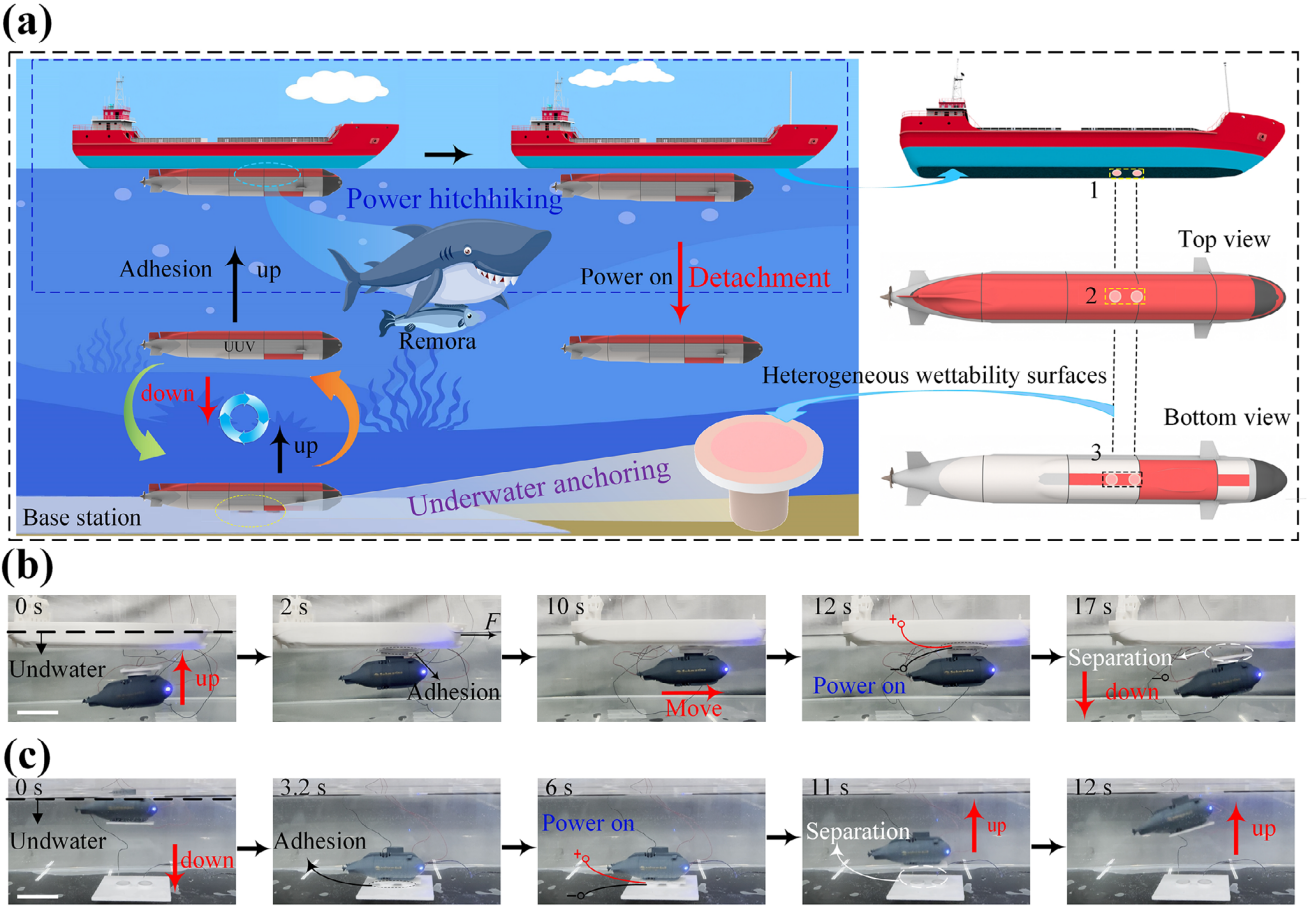
Applications. (a) Schematic diagram of the application of adhesives in the UUV. (b) Selected frames illustrating the adhesion and detachment processes between the UUV and the ship. Here, the UUV maintains a sinking state without propulsion via ballast weights. Scale bar: 6 cm. (c) Selected frames illustrating the adhesion and detachment processes between the UUV and the base station. Here, the UUV is in an ascending state without propulsion. Scale bar: 6 cm.

In view of these problems, we integrate capillary adhesion technology into the UUV systems. This approach enables two key operational modes: when performing long‐distance cruising missions, the UUV can adhere to the bottom of a moving ship (mimicking the survival strategy of remora) to achieve efficient power hitchhiking; for stationary monitoring tasks, it enables robust underwater anchoring, enhancing stability in complex environments (Figure 5a). As shown in Figure 5b and Movie S4, the UUV can easily adhere to the bottom of the ship and achieve stable hull‐towed motion under the drive of ascending propulsion. Likewise, under downward propulsion, it achieves robust adhesion to the base station (Figure 5c; Movie S5). Crucially, the electrically triggered adhesion failure enables on‐demand detachment from the ship or base station. Furthermore, the successful fabrication of heterogeneous wettability patterns on flexible aluminum substrates enables conformal adhesion on curved surfaces (Figure S15), significantly broadening the applicability of this technology across various underwater geometries and operational contexts. Thus, our design strategy provides a versatile and effective approach for UUV deployment across diverse underwater scenarios.

## 3 Conclusions

In this work, we develop a reversible underwater capillary adhesive based on a heterogeneous wettability surface design. This strategy enables robust adhesion and rapid on‐demand detachment between two surfaces through the formation of stable oil‐ring‐sealed water bridges, which generate a substantial Laplace pressure difference. Quantitative experiments confirm a remarkable adhesion strength of approximately 26.5 kPa between the heterogeneous surfaces, significantly outperforming the homogeneous surfaces. By constructing uniform arrays of alternating wettability, we achieve linear enhancement of adhesion strength with the number of oil‐water interfaces, reaching about 70 kPa for a 5‐array design, surpassing most existing capillary‐based adhesives. The established theoretical model accurately predicts the adhesion performance and broadens the design space for substrate dimensions. Furthermore, the adhesive exhibits excellent stability and long‐term durability, sustaining a 300 g load underwater for more than 72 h, and maintaining its performance over 50 adhesion‐detachment cycles. Finally, inspired by the survival strategy of the remora, we successfully integrate the adhesive into the UUV, demonstrating critical functionalities such as stable underwater anchoring and energy‐efficient hitchhiking on moving surfaces, with instant detachment triggered by electrical signals. This work provides a powerful and versatile strategy for underwater reversible adhesion and is expected to inject new vitality into the fields of marine engineering and underwater transportation.

## 4 Methods

### 4.1 Materials

Al sheets, Cu sheets, and Fe sheets of various sizes were purchased from Mingtai Aluminum Co., Ltd. (China). Paraffin liquid, silicone oil, and peanut oil were obtained from Aladdin and used without further purification. Fluoroalkylsilane [FAS, CF_3_(CF_2_)_5_(CH_2_)_2_Si(OCH_3_)_3_] was purchased from Degussa Co. Ltd (Germany). All other auxiliary devices (made of PLA, purchased from Shenzhen Zongwei Cube Technology Co., Ltd., China) were manufactured using a 3D printer (Shenzhen Zongwei Cube Technology Co., Ltd., China).

### 4.2 Fabrication of Heterogeneous Wettability Surfaces

The preparation process for heterogeneous wettability surfaces is shown in Figure S2. Taking the Al plate (radius *R* = 20 mm) as an example, first, the Al plate was polished with sandpaper (1500 grit) to remove the surface oxide layer and edge burrs. Then, the entire Al plate surface was etched using a nanosecond laser (SK‐CX30, SAKE Laser Technology Co., China, wavelength of 1064 nm, spot diameter of 10 µm, pulse duration of 100 ns, and maximum output power of 50 W) to create rough structures (etching speed of 200 mm/s, power of 18 W). Subsequently, the selected superhydrophobic regions were subjected to a secondary laser etching using identical parameters. After immersed in 1 wt.% FAS ethanol for 30 min, the samples were dried at 80°C for 10 min to obtain superhydrophobic surfaces. Finally, the superhydrophilic regions were etched using a laser again. After cleaning with deionized water, homogeneous wettability surfaces with consistent flatness were obtained.

### 4.3 Fabrication of Visualization Windows

First, ITO glass with a radius of 30 mm was cleaned with alcohol to remove surface impurities. After drying, the surface was treated by a cold plasma jet for 5 min to obtain a superhydrophilic surface. The surface was selectively masked and coated with NeverWet superhydrophobic spray. Finally, the surface mask was removed to obtain a surface with homogeneous wettability. The ITO glass and Al sheet were connected with a wire, and the electrolysis process of the internal water bridge could be observed after electrical activation. In addition, a visualization window (regular glass with a radius of 20 mm) used to observe the oil ring and water bridge was prepared using the same method as above.

### 4.4 Characterization

Adhesion force was characterized using a self‐assembled device (Figure S6), consisting of a vertical lifting platform, a force sensor (Guangzhou Simbatouch Electronic Technology Co., Ltd., China), sliders, buffer springs, and a clamping device. During testing, the upper sample was moved toward the lower fixed sample at a speed of 330 µm/s. When the preload force reached about 12 N, the upper sample was continuously raised until the two samples separated. The force sensor recorded the data during this process, which was transmitted to the computer via a graphical push‐pull force gauge. A 3D surface optical profiler (New View 9000, ZYGO, USA) was used to characterize the 3D morphology. The contact angle was measured using an optical contact angle meter (SL200KS, KINO, USA).

### 4.5 Adhesion Strength Analysis of Multiple Oil Rings

As shown in Figure 3b, when the number of oil rings is *n*, the adhesion force can be calculated as follows:

(10) $$\begin{array}{cc} F_{n} & =\sum_{i=1}^{n}F_{o,i}+\sum_{i=1}^{n}F_{w,i}\\ & =F_{o,1}+F_{w,1}+F_{o,2}+F_{w,2}+F_{o,3}\\ & \;+F_{w,3}+\cdots \cdots +F_{o,n}+F_{w,n} \end{array}$$

Since the width of each oil ring and water bridge is *R/2n*, the adhesion forces of oil ring 1 and water bridge 1 can be calculated using the following equations:

(11) $$F_{o,1}=\frac{2\gamma \pi R^{2}}{h}\left\{1-{\left[\frac{\left(2n-1\right)}{2n}\right]}^{2}\right\}\cos\theta_{1}$$

(12) $$\begin{array}{c} F_{w,1}=\frac{2\gamma \pi R^{2}}{h}\left\{{\left[\frac{\left(2n-1\right)}{2n}\right]}^{2}-{\left[\frac{\left(2n-2\right)}{2n}\right]}^{2}\right\}\left(\cos\theta_{1}+\cos\theta_{2}\right) \end{array}$$

The adhesion forces of oil ring 2 and water bridge 2 can be calculated as below:

(13) $$F_{o,2}=\frac{2\gamma \pi R^{2}}{h}\left\{{\left[\frac{\left(2n-2\right)}{2n}\right]}^{2}-{\left[\frac{\left(2n-3\right)}{2n}\right]}^{2}\right\}\left(2\cos\theta_{1}+\cos\theta_{2}\right)$$

(14) $$\begin{array}{cc} & F_{w,2}\\ & =\frac{2\gamma \pi R^{2}}{h}\left\{{\left[\frac{\left(2n-3\right)}{2n}\right]}^{2}-{\left[\frac{\left(2n-4\right)}{2n}\right]}^{2}\right\}\left(2\cos\theta_{1}+2\cos\theta_{2}\right) \end{array}$$

Similarly, the adhesion forces of oil ring *i* and water bridge *i* can be calculated as follows:

(15) $$\begin{array}{cc} & F_{o,i}\\ & =\frac{2\gamma \pi R^{2}}{h}\left\{\left[\frac{4n-4i+3}{4n^{2}}i\right]\cos\theta_{1}+\left[\frac{4n-4i+3}{4n^{2}}\left(i-1\right)\right]\cos\theta_{2}\right\} \end{array}$$

(16) $$\begin{array}{cc} & F_{w,i}\\ & =\frac{2\gamma \pi R^{2}}{h}\left\{\left[\frac{4n-4i+1}{4n^{2}}i\right]\cos\theta_{1}+\left[\frac{4n-4i+1}{4n^{2}}\left(i-1\right)\right]\cos\theta_{2}\right\} \end{array}$$

Accordingly, when the number of oil rings is *n*, the adhesion strength can be calculated by the following equation:

(17) $$\begin{array}{cc} \frac{F_{n}}{\pi R^{2}} & =\frac{\sum_{i=1}^{n}F_{o,1}+\sum_{i=1}^{n}F_{w,1}}{\pi R^{2}}\\ & =\frac{2\gamma }{h}\left[\left(\frac{3n+1+2n^{2}}{6n}\right)\cos\theta_{1}+\left(\frac{4n^{2}-1}{12n}\right)\cos\theta_{2}\right] \end{array}$$

The theoretical model provides guidance for practical applications and accurately explains the influence of each parameter on the adhesion force.

## Conflicts of Interest

The authors declare no conflicts of interest.

## Supporting information




**Supporting File 1**: advs74630‐sup‐0001‐SuppMat.docx.

**Supporting File 2**: advs74630‐sup‐0002‐MovieS1.mp4.

**Supporting File 3**: advs74630‐sup‐0003‐MovieS2.mp4.

**Supporting File 4**: advs74630‐sup‐0004‐MovieS3.mp4.

**Supporting File 5**: advs74630‐sup‐0005‐MovieS4.mp4.

**Supporting File 6**: advs74630‐sup‐0006‐MovieS5.mp4.

## Data Availability

The data that support the findings of this study are available from the corresponding author upon reasonable request.
