# Supplementary material for: Heterogeneous Wettability‐Based Construction of Two‐Phase Interfaces for Underwater Reversible Adhesion
Source: Adv Sci (Weinh). 2026 Mar 2;13(27):e24235. doi: 10.1002/advs.202524235 (PMC13170263; doi:10.1002/advs.202524235)
Supplement: Supplementary file 1 — Supporting File 1: advs74630‐sup‐0001‐SuppMat.docx. [file ADVS-13-e24235-s006.docx]

Supporting Information

Heterogeneous wettability-based construction of two-phase interfaces for underwater reversible adhesion

*Xiaokai Li*^a^, *Yonghui Zhang*^a^, *Yongxin Li*^a^, *Jiahao Zhang*^a^, *Yuheng Li*^a^, *Zhengyu Li*^a^, *Jianwu Wang*^a^, *Yaochen Lv*^a^, *Yanan Wang*^a^, *Jiyu Liu*^b,c*^, *Xin Liu*^a*^, *Huanxi Zheng*^a*^

^a^State Key Laboratory of High-performance Precision Manufacturing, Dalian University of Technology, Dalian 116024, P. R. China

^b^State Key Laboratory of Woody Oil Resources Utilization, Northeast Forestry University, Harbin 150040, P. R. China

^c^College of Mechanical and Electrical Engineering, Northeast Forestry University, Harbin, 150400, P. R. China

^*^Corresponding authors: [huanxizh@dlut.edu.cn](mailto:huanxizh@dlut.edu.cn); xinliu@dlut.edu.cn; liujiyu19940802@163.com

Content

**Note 1**: Calculation of the Adhesion Force

**Figure S1**. Stability comparison between air cavities and oil rings

**Figure S2**. Preparation process of heterogeneous wettability surface

**Figure S3** Related contact angle characterization.

**Figure S4**. Visualization of protective oil rings and encapsulated water bridges formed between two heterogeneous wettability surfaces

**Figure S5**. Selected optical photographs of superhydrophobic and superhydrophilic surfaces during motion carrying a 150 g load

**Figure S6**. Self-assembled device for testing adhesion forces

**Figure S7.** Surface morphology characterization

**Figure S8**. Optical picture of continuous adhesion of capillary adhesive in water

**Figure S9**. Oil phase distribution on heterogeneous wettability surfaces after multiple cycles

**Figure S10.** Adhesion properties under different aquatic environments

**Figure S11.** Changes in the oil ring underwater as a function of tim**e**

**Figure S12.** Adhesion force analysis

**Figure S13.** The comparison of adhesion strength between our work and other advanced adhesives

**Figure S14**. Electrolysis experiment of water bridge

**Figure S15**. Construction of different shapes of heterogeneous wettability patterns on flexible substrates

**Figure S16**. Schematic diagram of a water bridge between two superhydrophilic surfaces in air

**Movie1**. The underwater adhesion performance of samples with different surface wettability

**Movie2**. Electrically triggered fast on-demand pickup and release for moving 150 g weights from the water to the air

**Movie3**. The generation and growth of bubbles due to water bridge electrolysis

**Movie4**. The ship carries the UUV forward to achieve power hitchhiking

**Movie5**. The UUV enables on-demand adhesion to and detachment from the base station

**Supporting Information Note 1: Calculation of the Adhesion Force**

Here, taking the water bridge between two surfaces in air as an example (Fig. S16), the derivation process of capillary adhesion force between two heterogeneous surfaces is shown. The pressure difference (Laplace pressure difference) between the inner and outer surfaces of a water bridge is determined by surface tension and curvature radius. and can be calculated as $\Delta P=\gamma\left(1/R_{1}+1/R_{2}\right)$, where *R*_1_ is the transverse curvature radius of the water bridge (parallel to the plate surface direction, approximately equal to the contact radius *R*), and *R*_2_ is the axial curvature radius of the liquid bridge, which can be calculated as $R_{2}=h/2\cos\theta$.Thus, the Laplace pressure difference can be calculated as$\Delta P=\gamma\left(1/R+2\cos\theta/h\right)$.Then, the adhesion force contributed by the pressure difference can be obtained as. In addition, the adhesion component generated by surface tension can be calculated as $F_{\text{t}}=2\pi R\times\gamma\cos\theta$. Therefore, the total adhesion can be calculated as $F_{\text{total}}=F_{\text{p}}+F_{\text{t}}=\gamma\pi R+2\pi R^{2}\gamma\cos\theta/h+2\pi R\times\gamma\cos\theta$.Considering that $R\gg h$, the adhesion force can be simplified as $F_{\text{total}}\approx2\pi R^{2}\gamma\cos\theta/h$, and the corresponding adhesion strength can be calculated as $F_{\text{total}}/\pi R^{2}\approx2\gamma\cos\theta/h$.

Figure S1 illustrates the stability comparison between the air cavity and oil ring structures. Environmental disturbance tests (Figure S1a) demonstrate that the air cavity structure is highly susceptible to collapse in simulated underwater turbulent environments, leading to the escape of trapped gas (Figure S1b). In contrast, the oil ring structure remains stable throughout, unaffected by external environmental disturbances (Figure S1c). Durability experiments conducted in natural river water environments (Figure S1d) further reveal that the superhydrophobic surface has completely lost its hydrophobicity after 72 hours due to microbial fouling and pollutant deposition, rendering it incapable of sustaining the air cavity structure (Figure S1e). Conversely, the oil ring structure remains largely unaffected, consistently retaining its structural integrity and stability (Figure S1f).


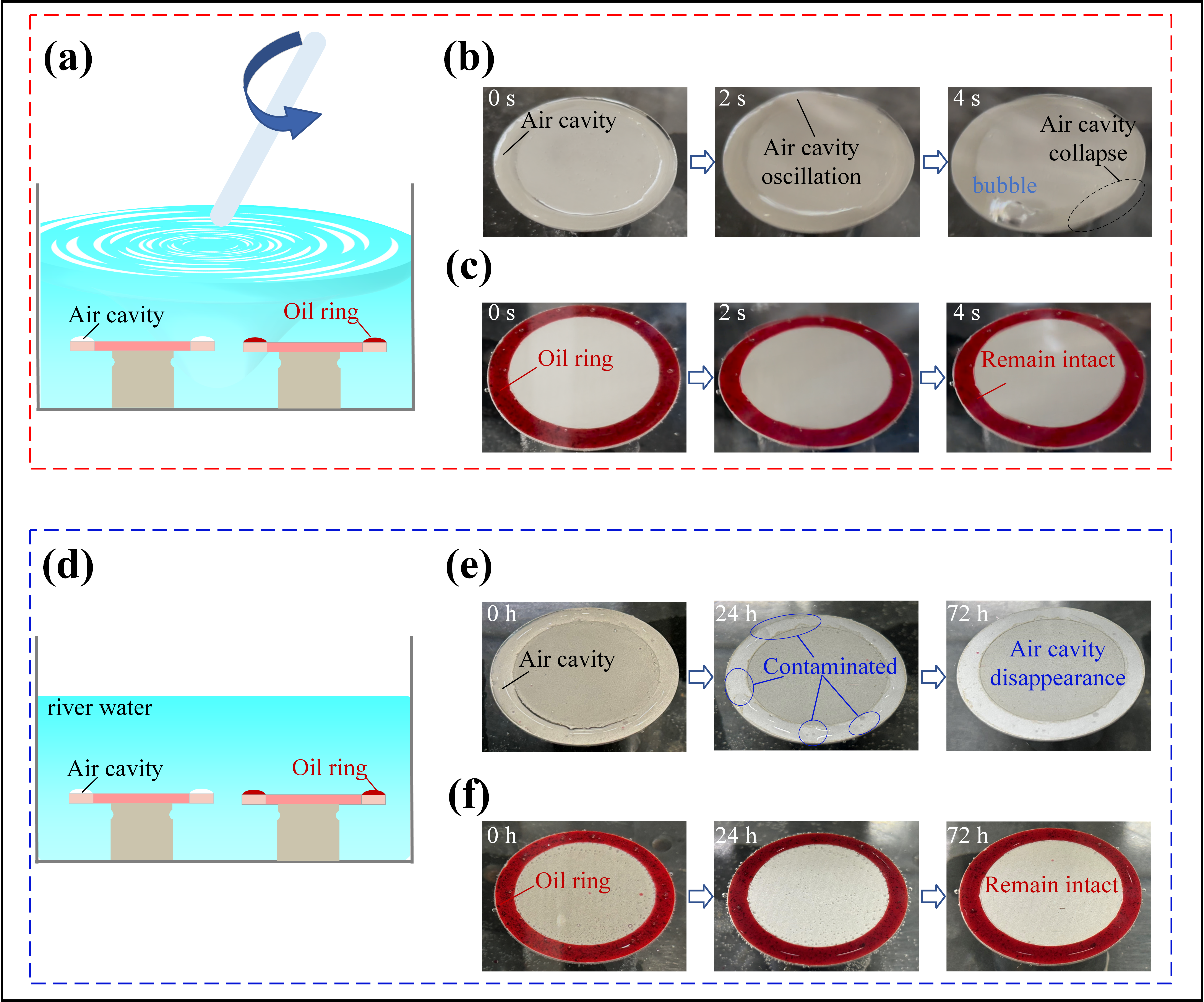


**Figure S1**. Stability comparison between air cavities and oil rings. (a) Schematic of interference resistance experiment. (b-c) Selected optical images demonstrate the evolution of air cavity (b) and oil ring (c) structures in simulated turbulent environments. (d) Schematic of durability experiment. (e-f) Effect of immersion time in natural river water on air cavities (e) and oil rings (f). Here, the oil rings are stained with oil-soluble fuel.


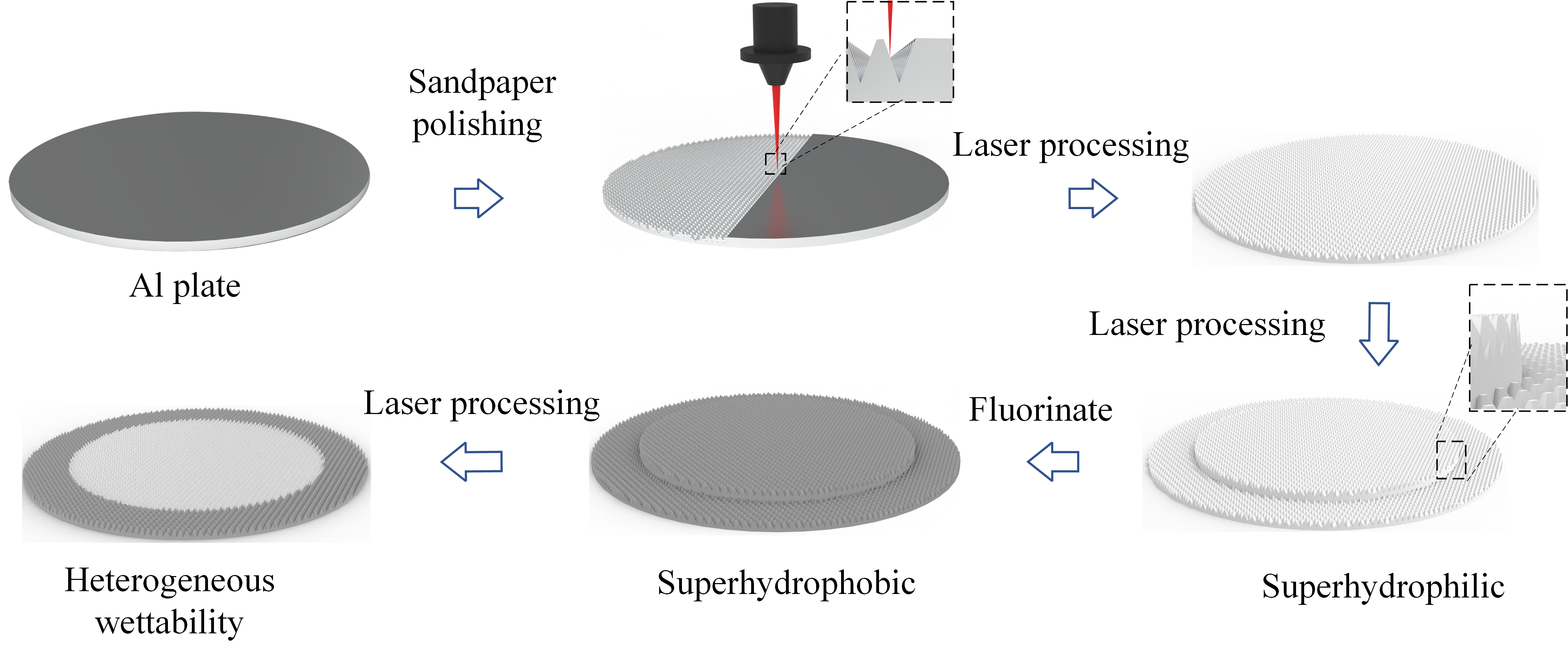


**Figure S2**. Preparation process of heterogeneous wettability surface. First, the Al plate is polished with sandpaper (1500 grit) to remove the surface oxide layer and edge burrs. Then, the entire Al plate surface is etched using a nanosecond laser to create a rough structure (etching speed of 200 mm/s, power of 18 W). Subsequently, the selected superhydrophobic regions are subjected to a second laser etching using the same parameters. After immersed in 1 wt.% FAS ethanol for 30 minutes, the sample is dried at 80°C for 10 minutes to obtain a superhydrophobic surface. Finally, the superhydrophilic regions are etched out using laser again. After cleaning with deionized water, a homogeneous wettability surface with consistent flatness is obtained.


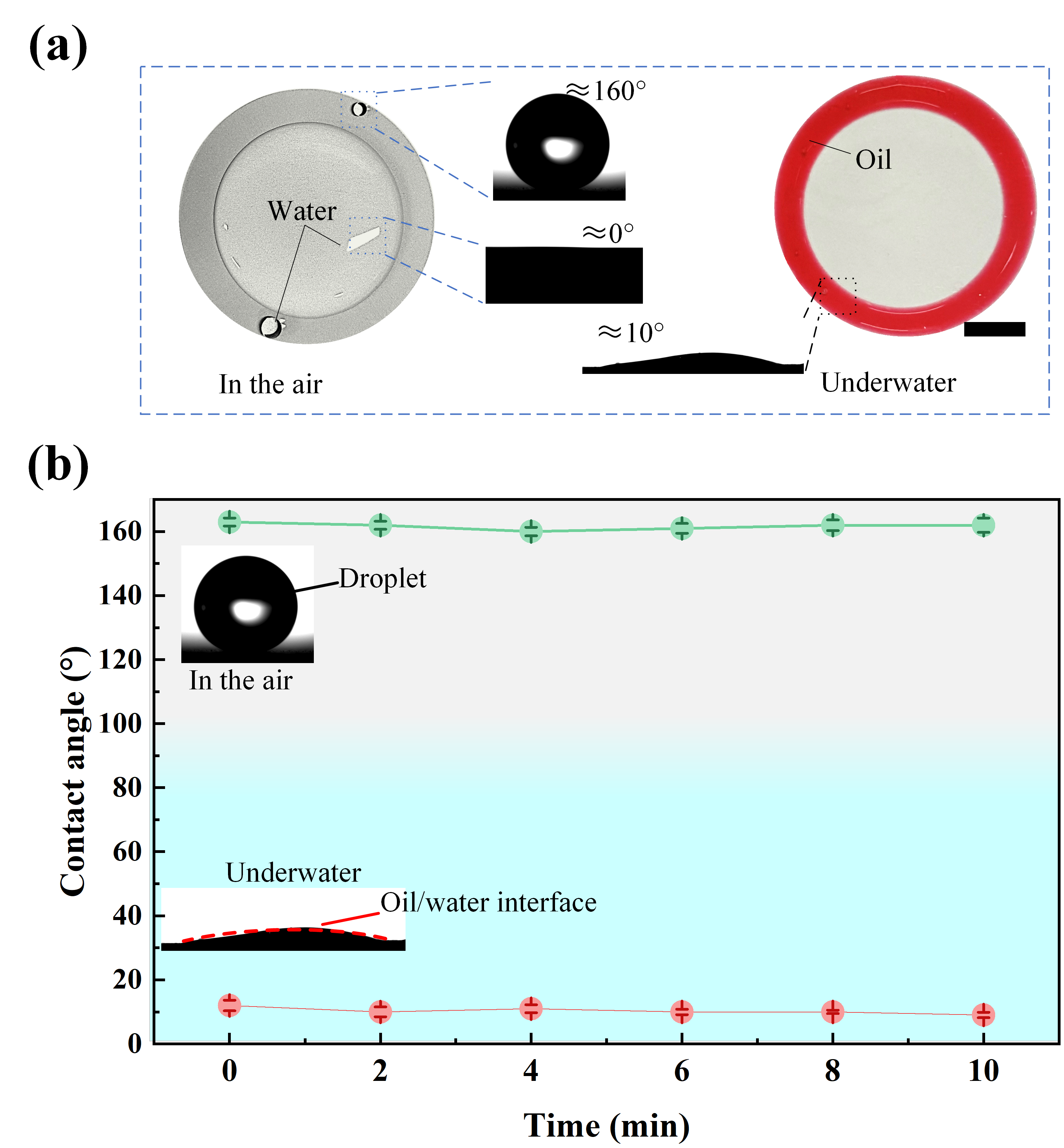


**Figure S3** Related contact angle characterization. (a) Optical images of the states of liquid droplets on heterogeneous wettability surfaces in air, and optical images of underwater air cavities after oil ring filling. (b) Contact angle evolution of droplets on a superhydrophobic surface, and that of oil on the same surface in an underwater environment. Here, the substrate is an Al sheet, and the oil phase is peanut oil. In air, the contact angle of droplets on the superhydrophobic surface remained essentially unchanged, consistently around 160°. Underwater, the contact angle of the oil phase on the same surface remained stable at approximately 10°.


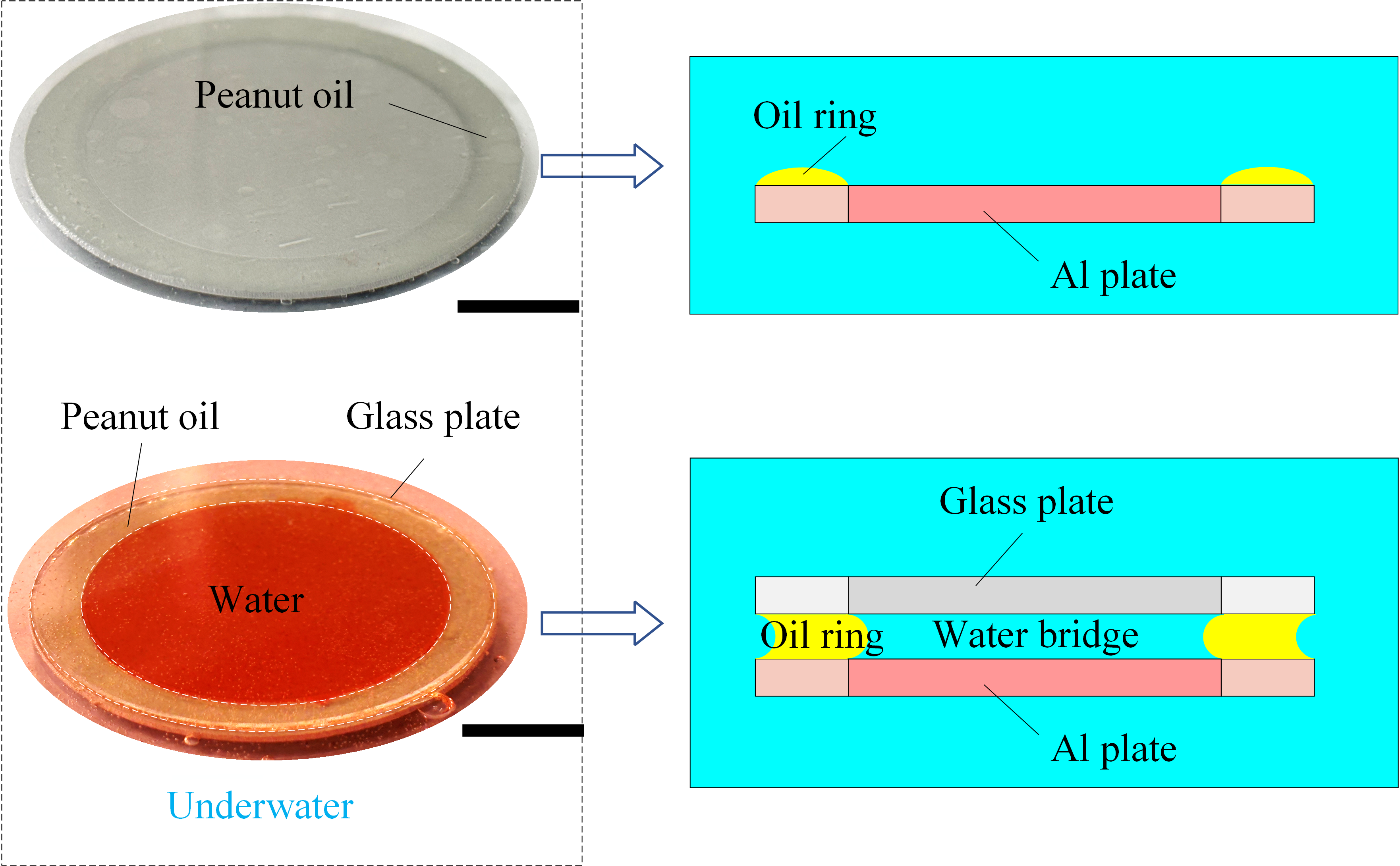


**Figure S4**. Visualization of protective oil rings and encapsulated water bridges formed between two heterogeneous wettability surfaces. Here, the scale bar is 1 cm, the filler is peanut oil, the bottom substrate is an Al sheet, and the visualization window at the top is a glass sheet with heterogeneous wettability. From air to underwater, the circular superhydrophobic region of the heterogeneous wettability surface forms a space-restricted air cavity, while the central superhydrophilic region is completely immersed in water. When the two heterogeneous wettability surfaces are brought into close proximity, excess oil/air mixture is extruded to form a thin water bridge and an intact oil ring, where the oil ring encapsulates the thin water bridge, thereby isolating it from the external water environment.


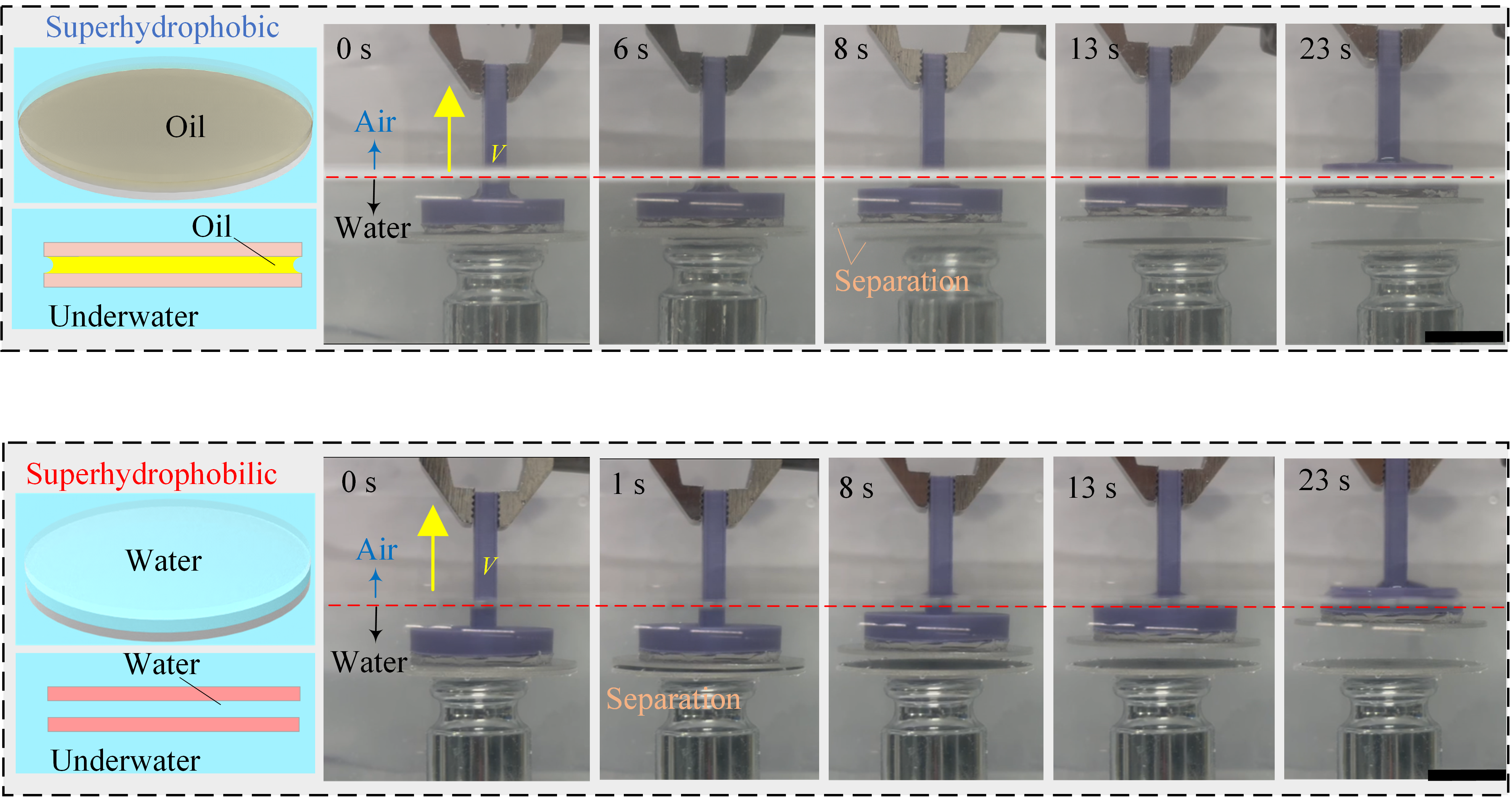


**Figure S5**. Selected optical photographs of superhydrophobic and superhydrophilic surfaces during motion carrying a 150 g load. Here, the scale bar is 1 cm, the substrate material is Al sheet, and the filler is paraffin liquid. The homogeneous superhydrophobic surface undergoes adhesion failure during load lifting, while the homogeneous superhydrophilic surface is completely wetted by water, resulting in oil-phase attachment failure, and no significant load-carrying motion is observed.


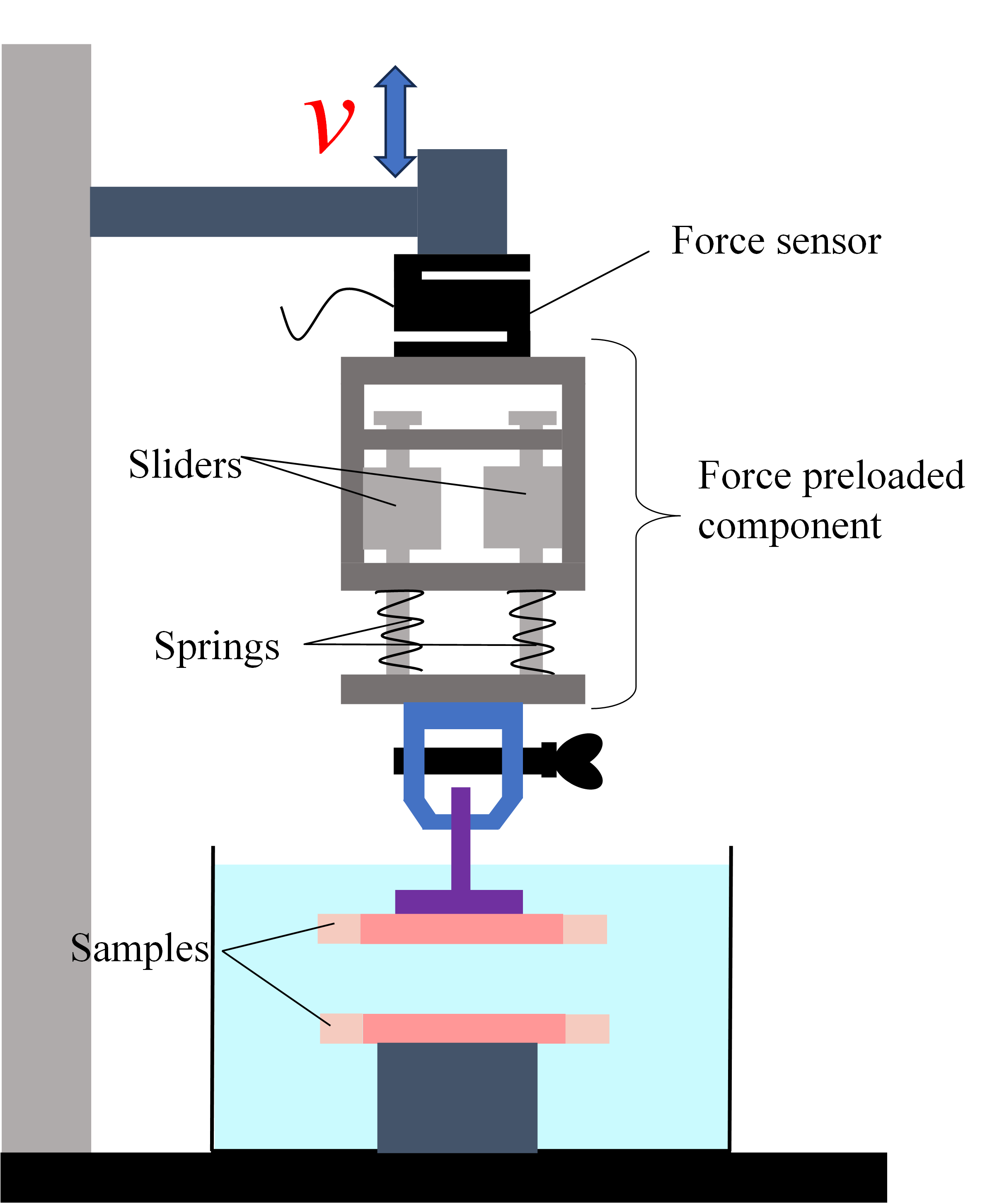


**Figure S6**. A self-assembled device for adhesion force testing. The device consists of a vertical lifting platform, a force sensor (Guangzhou Simbatouch Electronic Technology Co., Ltd., China), sliders, buffer springs, and clamping device. During the test, the top sample moves at a speed of 330 μm/s while the bottom sample remains fixed.


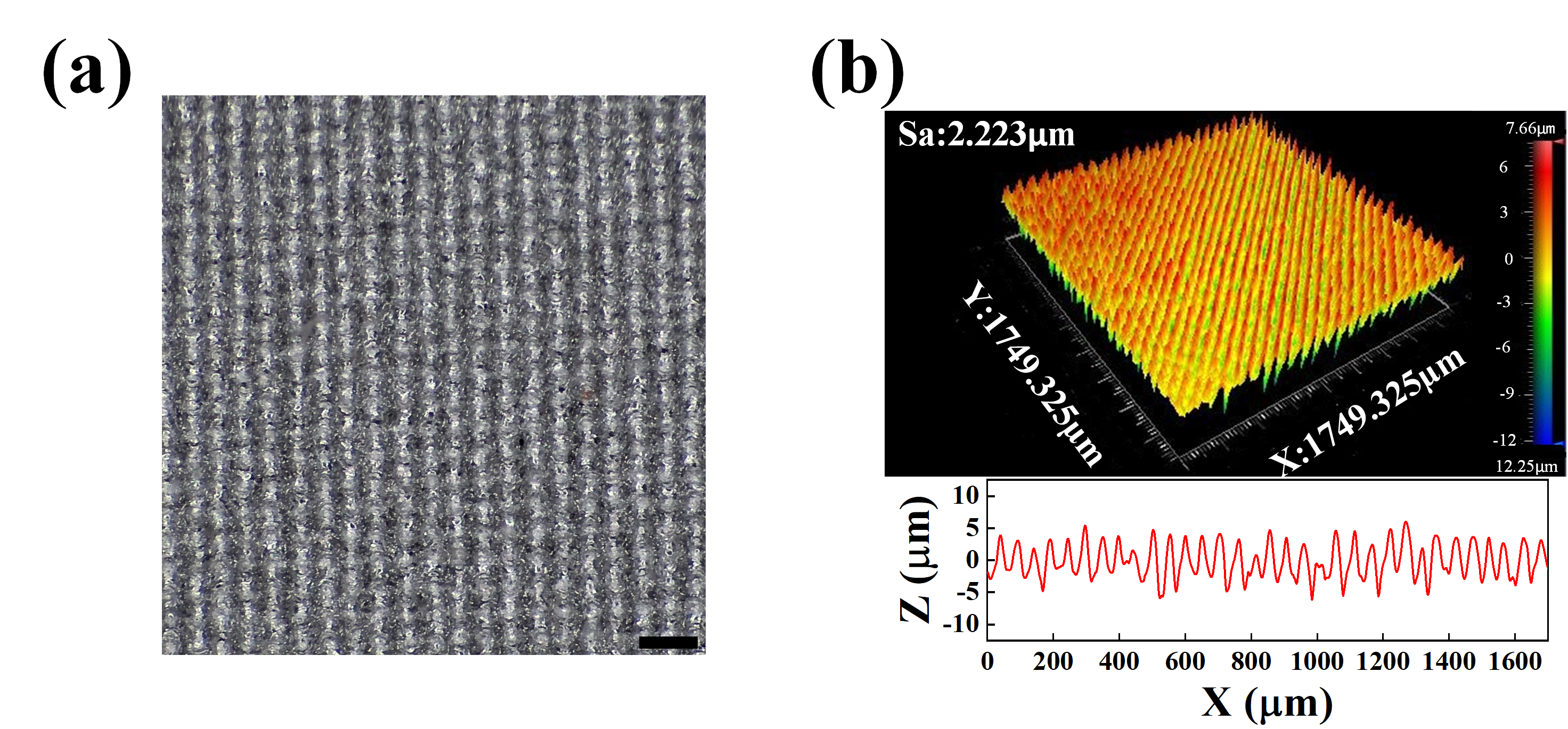


**Figure S7.** Surface morphology characterization. (a) Magnified surface morphology micrograph. Here, the scale bar is 0.1 mm. (b) 3D topography and cross-sectional data.


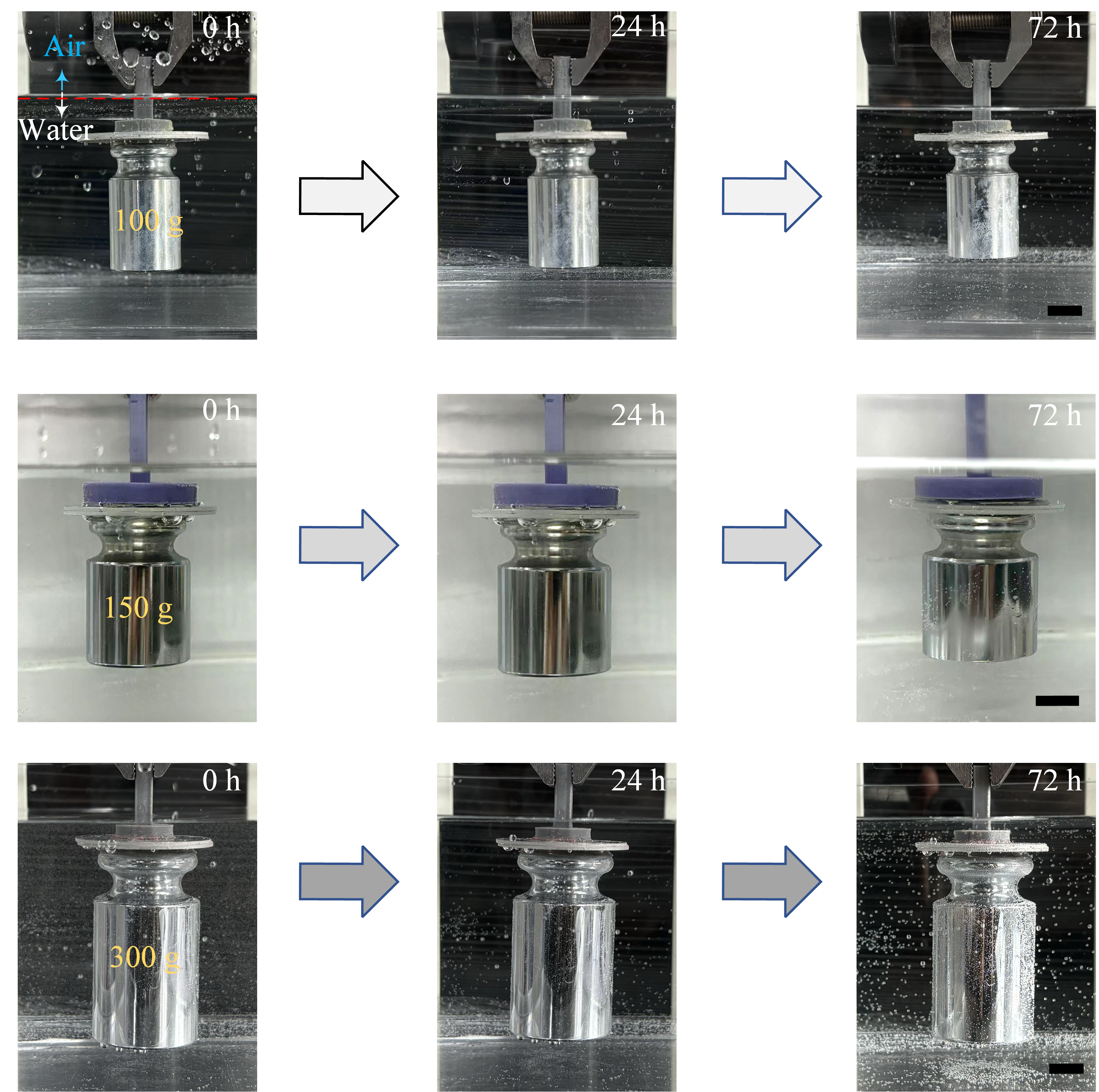


**Figure S8**. Optical image of continuous adhesion of the capillary adhesive in water. Here, the scale bar is 1 cm, the substrate material is Al sheet, and the filler is paraffin liquid. This capillary adhesive can maintain stable adhesion underwater with a load of 100–300g for more than 72 hours without failure. This fully demonstrates its excellent durability and scalability.


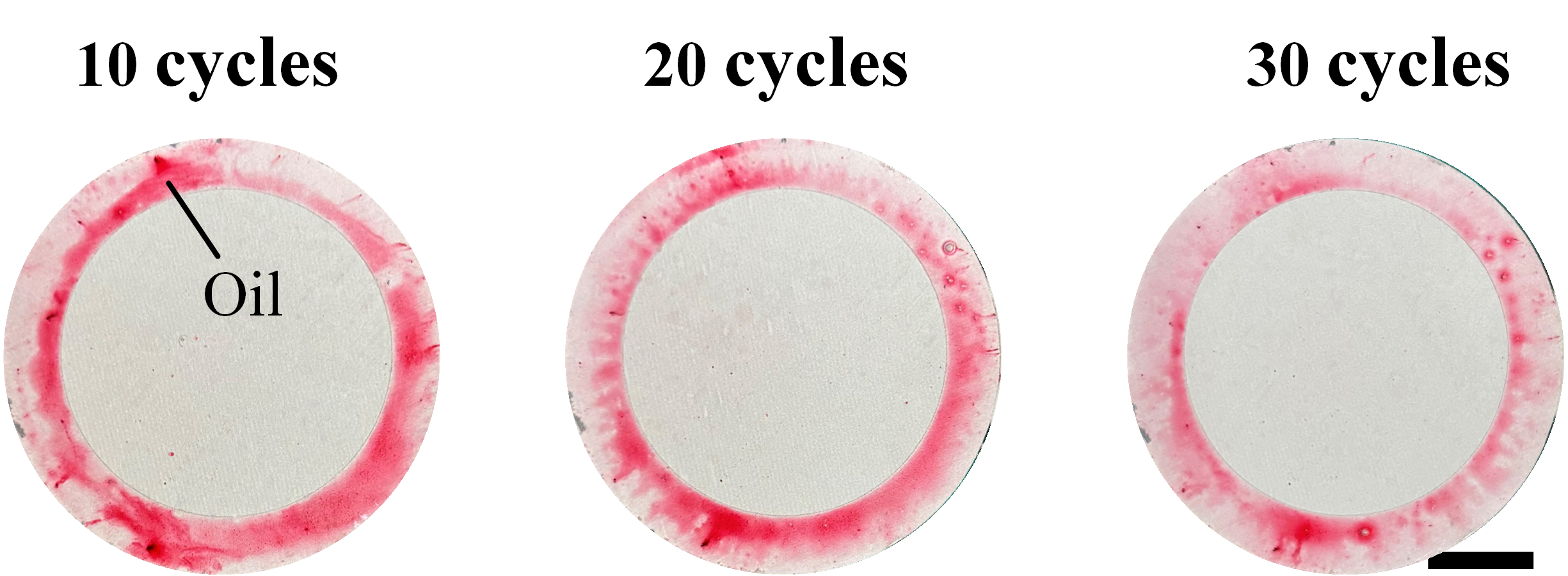


**Figure S9**. Oil phase distribution on heterogeneous wettability surfaces after multiple cycles. Here, the scale bar is 1 cm, the substrate is Al sheets, and the oil phase is stained peanut oil. Even after multiple attachment-detachment cycles, the oil phase on the superhydrophobic surface remains stably anchored without detachment or dispersion, effectively maintaining the integrity of its overall distribution structure.

**Figure S10.** Adhesion properties under different aquatic environments. Here, continuous stirring by the magnetic stirrer rotor drives the aqueous solution to form a stable flow field, simulating the dynamic aquatic environment encountered in practical applications. The samples were immersed in flowing water, natural rivers, and high-salinity water respectively for 6 hours before testing their adhesion to evaluate stability across different aquatic environments. Results showed no significant impairment of adhesion in any of these three underwater conditions, demonstrating our design's outstanding environmental stability.


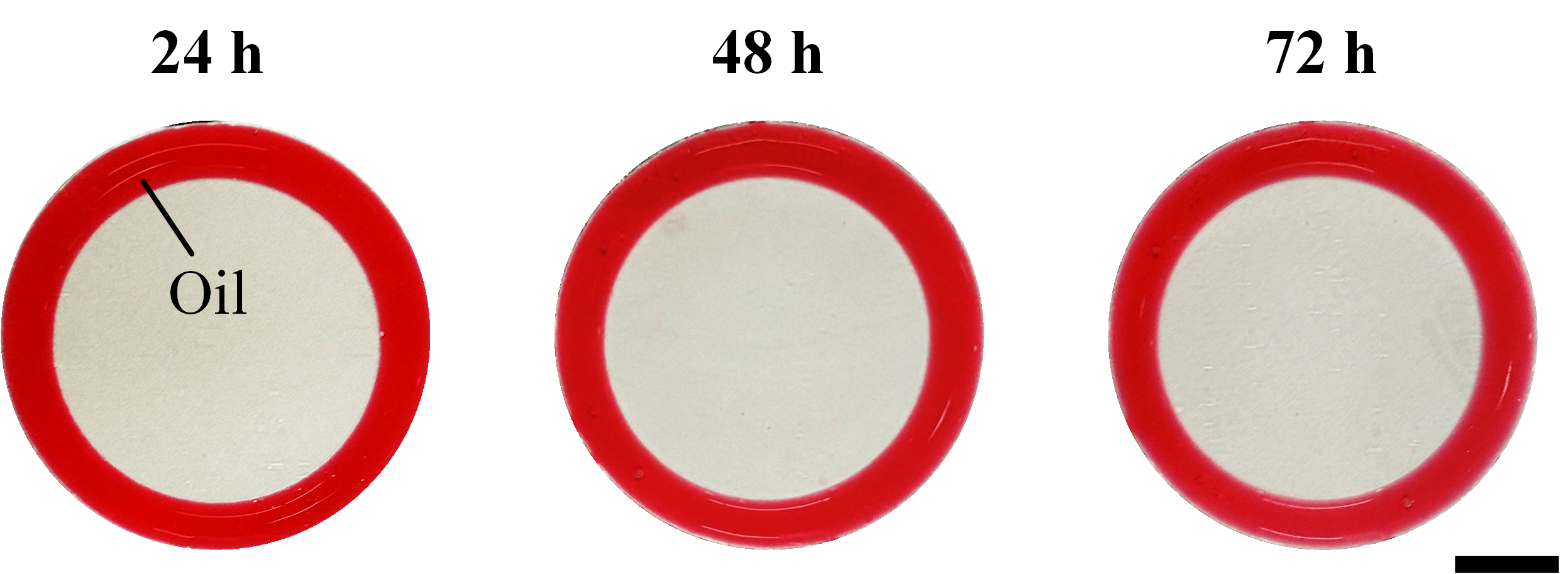


**Figure S11.** Changes in the oil ring underwater as a function of tim**e**. Here, the scale bar is 1 cm, the substrate is Al sheets, and the oil phase is stained peanut oil. As the immersion time increases, the oil ring remains anchored in the superhydrophobic region. Even after 12 hours of immersion, the oil ring retains its complete shape.


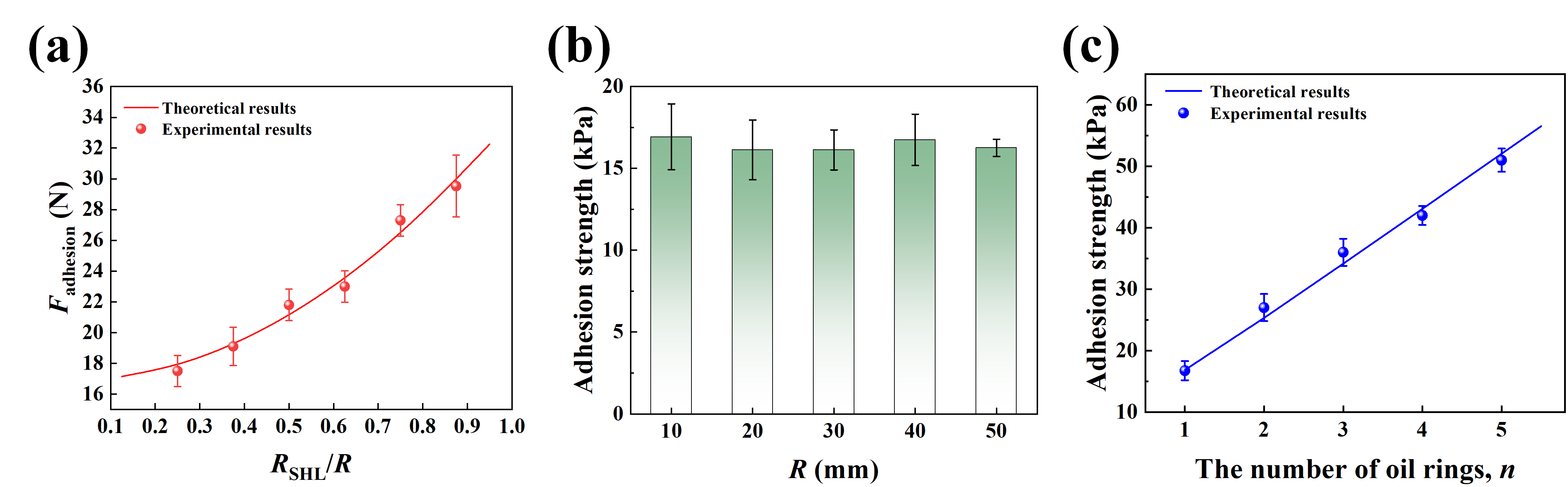


**Figure S12.** Adhesion force analysis. (a) The effect of $R_{\text{SHL}}/R$ on the adhesion strength of the experimental value and the mathematical model. Here, *R* = 2 cm. (d) The effect of *R* on adhesion strength. Here, $R_{\text{SHL}}/R$ is 0.5, and the adhesion strength is stable at ≈ 17 kPa (theoretical value). (e) The effect of the number of oil rings on the adhesion strength. The filler in (a) - (c) is peanut oil and the error bars are the deviations of five measurements. The experimental results closely match the mathematical model, confirming the accuracy of the mathematical model.

**Figure S13.** The comparison of adhesion strength between our work and other advanced adhesives. Here, the filler is paraffin liquid and the substrate is Al sheet. When *n* = 5, its adhesion force surpasses that of other adhesives currently prepared based on capillary effects. When *n* = 350, its adhesion force is as high as ≈ 4220 Kpa, not only exceeding that of adhesives currently prepared based on van der Waals forces, electrostatic forces, and suction forces, but also rivaling the most advanced chemically synthesized adhesives.


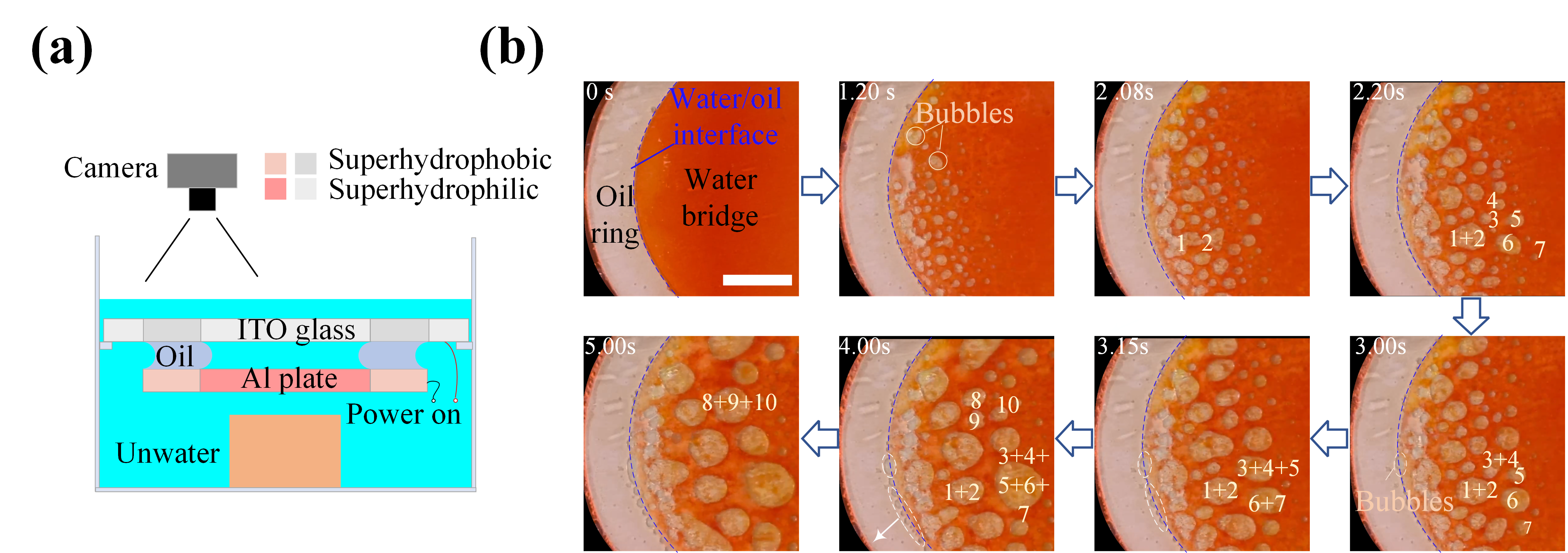


**Figure S14**. Electrolysis experiment of water bridge. (a) Schematic diagram of the experimental setup. (b) Selected images illustrating the electrolysis process of the internal water bridge. Here, the voltage is 30 V, the scale bar is 1 cm, and the visualization window at the top is an ITO glass plate with heterogeneous wettability. On one hand, the bubbles generated by the electrolysis of the water bridge reduce the contact area between the water bridge and the substrate; on the other hand, the continuous growth of the bubbles weakens the isolation effect of the oil ring.


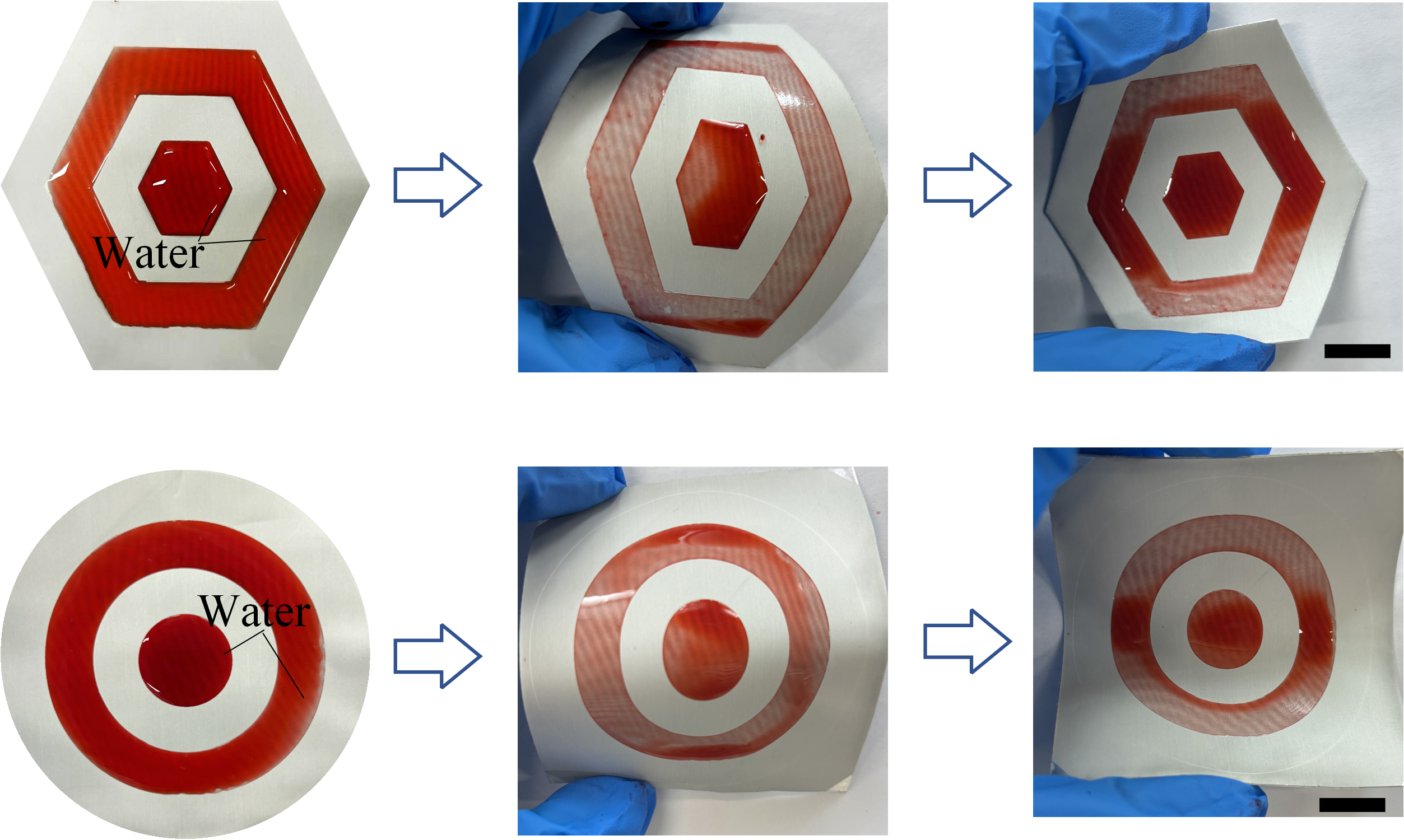


**Figure S15**. Construction of different shapes of heterogeneous wettability patterns on flexible substrates. The heterogeneous wettability surface constructed on flexible Al tape enables capillary adhesion between two concave-convex objects. Here, the thickness of the Al tape is 200 μm, *n* is 2, and the scale is 1 cm.


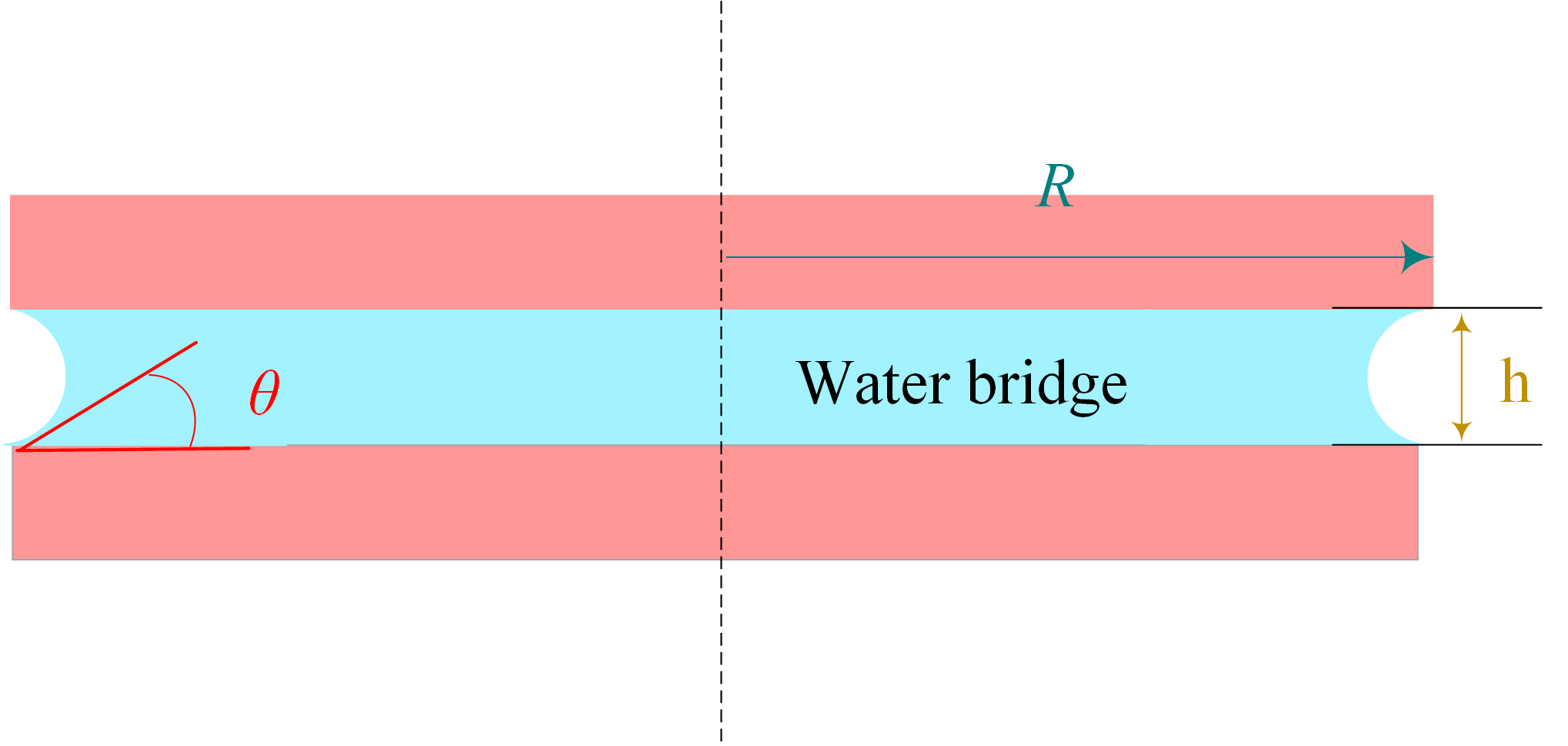


**Figure S16**. Schematic diagram of a water bridge between two superhydrophilic surfaces in air.

**Table S1**. Contact angle test summary

| Surface | Test Environment | Testing liquid | Contact angle |
| --- | --- | --- | --- |
| Superhydrophobic surface | In the air | Water | ≈160° |
|  | underwater | Peanut oil（after injecting the air cavity） | ≈10° |
|  |  | Paraffin Liquid（after injecting the air cavity） | ≈10° |
|  |  | Peanut oil（after injecting the air cavity） | ≈9° |
| Superhydrophilic surface | In the air | Water | ≈0° |
|  | underwater | Water | ≈0° |
|  |  | Peanut oil | Unable to attach |
|  |  | Paraffin liquid |  |
|  |  | Silicone oil |  |

**Table S2**. Switchable underwater adhesives summary

| Adhesion mechanism | Underwater adhesion | Detachment triggers | Release time | References |
| --- | --- | --- | --- | --- |
| Chemical bond | 2.7~21 kPa | Temperature change | 27 s | 33 |
|  | 50~270 kPa |  | ≈13 s | 29 |
|  | 0.2~10 kPa |  | ≈3600 s | 27 |
|  | 0.23~4 kPa |  | Within tens of seconds | 23 |
|  | 0.45~6.2 kPa |  |  | 34 |
| Pressure differential | 30kPa |  | 10s | 17 |
|  | 94 kPa |  | 5~30 s | 35 |
|  | ≈ 8.3kPa | Magnetic field | 50 s | 36 |
| Capillary force | 5.4kPa | Electrical stimulus | 6 s | 4 |
|  | 70kPa |  | 5 s | This work |

**References**

1. T. Weinstein, H. Gilon, O. Filc, C. Sammartino, B.-E. Pinchasik, *ACS Appl. Mater. Interfaces* **2022**, *14*, 9855.
2. Y. Li, R. Wang, S. Jiao, H. Lai, Y. Liu, Z. Cheng, *Chemical Engineering Journal* **2023**, *461*, 141927
3. M. J. Vogel, P. H. Steen, *Proceedings of the National Academy of Sciences* **2010**, *107*, 3377.
4. H. Zheng, J. Li, Y. Zhou, C. Zhang, W. Xu, Y. Deng, J. Li, S. Feng, Z. Yi, X. Zhou, X. Ji, P. Shi, Z. Wang, *Nat Commun* **2022**, *13*, 4584.
5. Z. Ma, L. Liang, C. Zhang, Y. Xiang, M. Yan, Z. Liu, W. Wang, S. Yan, J. Zhao, *ACS Appl. Mater. Interfaces* **2025**, *17*, 20471.
6. Z. Ye, G. Z. Lum, S. Song, S. Rich, M. Sitti, *Advanced Materials* **2016**, *28*, 5088.
7. E. Kizilkan, S. N. Gorb, *ACS Appl. Mater. Interfaces* **2018**, *10*, 26752.
8. K. Jin, J. C. Cremaldi, J. S. Erickson, Y. Tian, J. N. Israelachvili, N. S. Pesika, *Advanced Functional Materials* **2014**, *24*, 574.
9. Y. Wang, X. Zhang, R. Hensel, E. Arzt, *Advanced Materials Interfaces* **2022**, *9*, 2101764.
10. J. Zhao, X. Li, Y. Tan, X. Liu, T. Lu, M. Shi, *Advanced Materials* **2022**, *34*, 2107748.
11. H. E. Jeong, M. K. Kwak, K. Y. Suh, *Langmuir* **2010**, *26*, 2223.
12. H. J. Kim, L. Paquin, C. W. Barney, S. So, B. Chen, Z. Suo, A. J. Crosby, R. C. Hayward, *Advanced Materials* **2020**, *32*, 2000600.
13. K. Choi, Y. Chan Kim, H. Sun, S.-H. Kim, J. W. Yoo, I.-K. Park, P.-C. Lee, H. J. Choi, H. R. Choi, T. Kim, J. Suhr, Y. K. Lee, J.-D. Nam, *ACS Omega* **2019**, *4*, 7994.
14. L. K. Borden, A. Gargava, S. R. Raghavan, *Nat Commun* **2021**, *12*, 4419.
15. M. Tatari, A. Mohammadi Nasab, K. T. Turner, W. Shan, *Advanced Materials Interfaces* **2018**, *5*, 1800321.
16. H. Xu, F. Yang, Y. Zhang, X. Jiang, L. Wen, *IEEE Robotics and Automation Letters* **2022**, *7*, 3547.
17. Y.-W. Lee, S. Chun, D. Son, X. Hu, M. Schneider, M. Sitti, *Advanced Materials* **2022**, *34*, 2109325.
18. H. Luo, S. Wang, C. Wang, C. Linghu, J. Song, *Advanced Functional Materials* **2021**, *31*, 2010297.
19. H. J. Lee, S. Baik, G. W. Hwang, J. H. Song, D. W. Kim, B. Park, H. Min, J. K. Kim, J. Koh, T.-H. Yang, C. Pang, *ACS Nano* **2021**, *15*, 14137.
20. S. H. Lee, H. W. Song, B. S. Kang, M. K. Kwak, *ACS Appl. Mater. Interfaces* **2019**, *11*, 47571.
21. Y. Wang, V. Kang, W. Federle, E. Arzt, R. Hensel, *Advanced Materials Interfaces* **2020**, *7*, 2001269.
22. N. Bayat, Y. Zhang, P. Falabella, R. Menefee, J. J. Whalen, M. S. Humayun, M. E. Thompson, *Science Translational Medicine* **2017**, *9*, eaan3879.
23. Y. Zhao, Y. Wu, L. Wang, M. Zhang, X. Chen, M. Liu, J. Fan, J. Liu, F. Zhou, Z. Wang, *Nat Commun* **2017**, *8*, 2218.
24. Y. Ma, S. Ma, Y. Wu, X. Pei, S. N. Gorb, Z. Wang, W. Liu, F. Zhou, *Advanced Materials* **2018**, *30*, 1801595.
25. X. Liu, Q. Zhang, L. Duan, G. Gao, *Advanced Functional Materials* **2019**, *29*, 1900450
26. Z. Wang, J. Zhao, W. Tang, T. He, S. Wang, X. He, Y. Chen, D. Yang, S. Peng, *ACS Appl. Mater. Interfaces* **2021**, *13*, 3435
27. H. Abe, D. Yoshihara, S. Tottori, M. Nishizawa, *NPG Asia Mater* **2024**, *16*, 49
28. Y. Sun, Q. Li, W. Peng, C. Cai, F. Tang, Y. Liu, Q. Hu, J. Wang, B. Luo, X. Li, S. Nie, *Nano Lett.* **2025**, *25*, 6461
29. D. Tan, F. Meng, Y. Ni, W. Sun, Q. Liu, X. Wang, Z. Shi, Q. Zhao, Y. Lei, S. Luan, L. Xue, *Chemical Engineering Journal* **2023**, *471*, 144625.
30. C. Cui, C. Fan, Y. Wu, M. Xiao, T. Wu, D. Zhang, X. Chen, B. Liu, Z. Xu, B. Qu, W. Liu, *Advanced Materials* **2019**, *31*, 1905761
31. Z. Wang, L. Guo, H. Xiao, H. Cong, S. Wang, *Mater. Horiz.* **2020**, *7*, 282
32. Y. Liu, K. Li, J. Tian, A. Gao, L. Tian, H. Su, S. Miao, F. Tao, H. Ren, Q. Yang, J. Cao, P. Yang, *Nat Commun* **2023**, *14*, 5145.
33. C. Zhao, L. Chen, Y. Ru, L. Zhang, M. Liu, *Soft Matter* **2022**, *18*, 5934.
34. A. Eklund, O. Ikkala, H. Zhang, *Advanced Functional Materials* **2024**, *34*, 2214091.
35. H. Lee, D.-S. Um, Y. Lee, S. Lim, H. Kim, H. Ko, *Advanced Materials* **2016**, *28*, 7457.
36. S. Wang, H. Luo, C. Linghu, J. Song, *Advanced Functional Materials* **2021**, *31*, 2009217.
